# NAT10-mediated ac4C modification of KDM1B drives osteoarthritis progression through epigenetic suppression of SOX9

**DOI:** 10.1007/s00018-025-05918-z

**Published:** 2025-11-26

**Authors:** Shuxiang Chen, Wenhuan Ou, Xiaotao Li, Mufu Jie, Yi Zheng, Jian Situ, Zhipeng Liao, Li Huang, Weizhong Qi, Songjia Ni

**Affiliations:** 1 Department of Orthopaedic, Jiangmen Wuyi Hospital of Traditional Chinese Medicine, Jiangmen, Guangdong China; 2 Department of Orthopaedic, Enping Branch of Jiangmen Wuyi Hospital of Traditional Chinese Medicine, Jiangmen, Guangdong China; 3 Department of Orthopaedic, Zhujiang Hospital, Southern Medical University, Guangzhou, Guangdong China

**Keywords:** Osteoarthritis, KDM1B, NAT10, N4-acetylcytidine, SOX9, Cartilage degradation

## Abstract

Histone methylation acts as a crucial regulator of diverse pathophysiological processes in humans. However, the involvement of histone methylation modification enzymes in osteoarthritis (OA) remains poorly characterized. Here, we delineated lysine demethylase 1B (KDM1B) as a nodal epigenetic effector driving OA pathobiology through an integrated strategy that combined data mining, bioinformatics analysis, and experimental validation. Gain-of-function studies revealed that chondrocyte - specific KDM1B overexpression amplified IL-1β-induced chondrocyte injury primarily through the inhibition of SRY-box transcription factor 9 (SOX9). Conversely, KDM1B knockdown inhibited IL-1β-induced chondrocyte damage in vitro and significantly alleviated OA progression in vivo by upregulating SOX9. Mechanistically, NAT10-catalyzed ac4C epitranscriptomic editing to KDM1B mRNA stabilization, leading to its overexpression in osteoarthritic chondrocytes. Elevated KDM1B subsequently binds to the promoter region of the SOX9 and catalyzes the demethylation of H3K4me2 to suppress SOX9 expression in osteoarthritic chondrocytes. Furthermore, our data also demonstrated that chondrocyte-specific NAT10 knockdown attenuated IL-1β-induced chondrocyte injury by modulating the KDM1B/SOX9 axis. Collectively, our findings unveil a novel NAT10/KDM1B/SOX9 regulatory cascade in OA pathogenesis, highlighting the potential of targeting epigenetic regulators to restore chondrocyte function and mitigate OA progression.

**Supplementary Information:**

The online version contains supplementary material available at 10.1007/s00018-025-05918-z.

## Introduction

Osteoarthritis (OA), a degenerative joint condition characterized by progressive cartilage degradation and functional impairment, affects over 500 million individuals globally according to recent epidemiological data [1]. Contemporary osteoarthritis management, encompassing pharmacological interventions, regenerative therapies, and joint replacement surgery, demonstrates therapeutic limitations by primarily achieving symptomatic relief rather than arresting the underlying biological mechanisms driving disease progression [2]. As the exclusive cellular components of articular cartilage, chondrocytes govern tissue homeostasis through precise regulation of extracellular matrix (ECM) biosynthesis and proteolytic remodeling [3]. Recent studies have highlighted that dysregulated chondrocytic proliferation and aberrant matrix turnover kinetics constitute fundamental pathomechanisms in OA development [4, 5]. However, the molecular mechanisms driving chondrocytes dysfunction in OA are still not fully elucidated. Consequently, elucidating these mechanisms is essential to pinpoint actionable therapeutic targets that can facilitate disease-modifying interventions, instead of focusing merely on symptomatic relief.

Epigenetics refers to the phenomenon of regulating gene expression and cell identity through heritable mechanisms without changing DNA sequence, such as DNA methylation, histone modifications, chromatin remodeling, and non-coding RNAs. Among these, histone methylation has emerged as a key regulator in OA initiation and progression, offering potential therapeutic targets [6–8]. KDM1B, a member of the KDM1 family of histone demethylases, specifically targets histone 3 lysine 4 (H3K4) for demethylation [9]. Growing evidence indicates that KDM1B plays crucial functions in multiple biological processes and disease progression. For example, KDM1B involves in the malignant progression of multiple cancers, positioning it as a promising target for epigenetic cancer therapy [9, 10]. Additionally, KDM1B regulates oocyte development [11] and promotes tear secretion dysfunction in Sjogren’s syndrome through the PAX6/CLU axis [12]. Despite these findings, the functions and underlying mechanisms of KDM1B in OA progression remain unexplored.

Post-transcriptional mRNA modifications, catalyzed by specialized and often conserved enzyme complexes, are essential for gene expression regulation, and dysregulation of these modifications can contribute to the development of various diseases [13]. N4-acetylcytidine (ac4C), an evolutionarily conserved RNA modification, enhances translation efficiency and mRNA stability by enriching cytosine bases at wobble positions [14]. N-acetyltransferase 10 (NAT10), the only known human enzyme with both acetyltransferase and RNA-binding activities, is responsible for ac4C modification and has been implicated in the pathology of numerous diseases [15]. Emerging evidence suggests that NAT10-mediated ac4C modification regulates mRNA stability and contributes to the development of inflammatory, metabolic, autoimmune, and neoplastic diseases [16]. However, the role of NAT10 and ac4C modification in OA remains poorly understood.

This study elucidates a novel regulatory mechanism of histone demethylase KDM1B in OA pathogenesis. Experimental evidence demonstrates that NAT10-mediated ac4C modification stabilizes KDM1B mRNA, leading to its overexpression in osteoarthritic chondrocytes. Significantly, shRNA-mediated KDM1B knockdown effectively attenuates IL-1β-induced chondrocytes injury in vitro and alleviates OA progression in vivo by suppressing SOX9. These findings not only offer new insights into OA pathogenesis but also establish the NAT10/ac4C/KDM1B/SOX9 signaling axis as a promising target for innovative OA treatments.

## Materials and methods

### Data sources

To identify differentially expressed genes (DEGs) between normal and OA cartilage tissues, transcriptomic comparisons of osteochondral homeostasis were conducted using publicly available RNA-seq datasets retrieved from the Gene Expression Omnibus (GEO). These datasets were systematically processed and analyzed through bioconductor packages in the R statistical environment to ensure robust identification of DEGs associated with OA pathogenesis. The GSE178557 dataset (https://www.ncbi.nlm.nih.gov/geo/query/acc.cgi?acc=GSE178557, 4 normal cartilage samples vs. 4 OA cartilage samples) and the GSE168505 dataset (https://www.ncbi.nlm.nih.gov/geo/query/acc.cgi?acc=GSE168505, 3 normal cartilage samples vs. 4 OA cartilage samples) were employed to identify candidate genes that regulate OA progression. The GSE114007 dataset (https://www.ncbi.nlm.nih.gov/geo/query/acc.cgi?acc=GSE114007), which includes 18 normal and 20 OA cartilage tissues, was used as an independent validation cohort to confirm reproducibility of candidate genes.

### Clinical sample acquisition

Human articular cartilage specimens were aseptically collected from patients undergoing total knee replacement surgery for knee OA at Zhujiang Hospital of Southern Medical University (Guangzhou, China). Following surgical extraction, a portion of the freshly obtained from cartilage specimens were immediately used for the isolation and culture of primary chondrocytes, whereas the residual samples were promptly frozen in liquid nitrogen and then preserved at −80 °C for subsequent biomolecular analyses. All patient’s inclusion adhered to standardized diagnostic protocols established by the American College of Rheumatology [17]. The extent of cartilage damage was quantitatively assessed through histopathological examination the Modified Outerbridge Classification, conducted by certified histopathologists. This investigation received ethical clearance from the Institutional Research Ethics Committee of Zhujiang Hospital of Southern Medical University (Approval Number: 2018-FSMYK-001), with full compliance to Declaration of Helsinki principles. Written informed consent was systematically procured from all participants (Age range: 58–75 years) during preoperative consultations, ensuring comprehensive understanding of specimen usage protocols.

### Primary chondrocyte isolation and OA modeling in vitro

Human primary chondrocytes were extracted from articular cartilage harvested from OA patients undergoing total knee arthroplasty following previously described protocols [18]. The extracted chondrocytes were expanded in growth medium consisting of DMEM/F12 medium (Thermo Fisher Scientific) supplemented with 10% fetal bovine serum (FBS, Gibco) under standard culture conditions (37 °C, 5% CO₂, 95% humidity). To ensure optimal cell growth conditions, complete medium replacement was conducted every 24 h. Second-passage (P2) chondrocytes were selected for experiments to preserve phenotypic stability and reduce the likelihood of dedifferentiation during subsequent analyses. An in vitro model of osteoarthritic chondrocyte injury was established by stimulating the human primary chondrocytes with interleukin-1β (IL-1β, Sigma-Aldrich) at varying concentrations spanning from 0 to 10 ng/mL for a duration of 24 h.

### Adenovirus production and infection efficiency

Adenoviruses containing short hairpin RNA (adv-shRNA) targeting KDM1B, NAT10, and SOX9, alongside adenoviruses designed for the overexpression of these genes, were procured from Aiji Biotechnology (Guangzhou, Guangdong, China). The infection of chondrocytes with these adenoviruses was executed in accordance with the manufacturer’s recommended protocol. At 24 h post-infection, the infection efficiency was validated through real-time quantitative PCR (RT-qPCR) analysis. The specific sequences of all shRNAs and their respective negative controls are detailed in Supplementary Table 1.

### Cell proliferation

Cell proliferation was evaluated utilizing the Cell Counting Kit-8 (CCK-8) and 5-ethynyl-2’-deoxyuridine (EdU) assays. For the CCK-8 assay, chondrocytes were processed with the Enhanced Cell Counting Kit-8 (Beyotime, C0042) in strict compliance with the manufacturer’s guidelines. Optical density was measured at 450 nm by a microplate reader (Tecan, F50). In the case of the EdU assay, chondrocytes were subjected to the BeyoClick™ EdU Cell Proliferation Kit with Alexa Fluor 488 (Beyotime, C0071) as per the specified protocol. Nuclear counterstaining was executed with Hoechst 33,342, and visual documentation was acquired through an inverted fluorescence microscope (Mshot, MF52). The acquired images underwent analysis employing ImageJ 1.46r software (U.S. National Institutes of Health, Bethesda, Maryland, USA), and the extent of cell proliferation was quantified as the ratio of EdU-positive cells to the entire cell population (Hoechst 33342-stained nuclei), presented as (EdU-positive cells × 100%)/total cells.

### RT-qPCR analysis

Total RNA was harvested from cartilage tissues and chondrocytes utilizing TRIzol reagent (Takara, Japan), followed by reverse transcription into cDNA with the PrimeScript™ RT Reagent Kit with gDNA Eraser (RR047A, Takara) in strict compliance with the manufacturer’s instructions. The RT-qPCR amplification was carried out employing the LightCycler^®^ 96 platforn (Roche) with BenyoFast™ SYBR Green qPCR Mix (D7260, Beyotime) in accordance with the manufacturer’s protocols. Gene expression levels were standardized relative to the geometric mean of two reference genes, HPRT1 and GAPDH, which were chosen due to their consistent expression in chondrocytes throughout the experimental procedures. The relative mRNA expression levels of target genes were calculated using the 2^−∆∆Ct^ method, and primer sequences for both target genes and reference genes are listed in Supplementary Table 2.

### Western blot analysis

Total protein was lysed from the specified cartilage tissues and chondrocytes using a pre-cooled RIPA lysis buffer (Solarbio) containing protease/phosphatase inhibitor cocktail. Protein concentrations were quantified using a Bicinchoninic Acid (BCA) Detection Kit (Beyotime, China) against BSA standard curves with λ = 562 nm absorbance readings. Identical quantities of protein samples were separated by mean of sodium dodecyl sulfate-polyacrylamide gel electrophoresis (SDS-PAGE) and subsequently transferred to polyvinylidene fluoride (PVDF) membranes (Millipore, USA) by electrophoretic transfer. Following blocking with 5% bovine serum albumin (BSA), the membranes were subjected to overnight incubation with primary antibodies at 4 °C. Subsequently, they were incubated with HRP-conjugated secondary antibodies for 2 h at room temperature. Ultimately, protein bands were visualized employing an enhanced chemiluminescence (ECL) reagent (DINGGUO Biology, China) on a gel imaging system (GE Healthcare) under dynamic exposure optimization, and band densitometry was normalized to β-actin loading controls utilizing ImageJ 1.46r software (U.S. National Institutes of Health, Bethesda, Maryland, USA) with rolling ball background subtraction. Comprehensive details regarding all antibodies employed in this study are furnished in Supplementary Table 3.

### Establishment and therapeutic intervention of mouse OA model

Male C57BL/6 mice (SPF, 12 weeks old) were sourced from the Laboratory Animal Center of Southern Medical University (Guangzhou, China). All animal experiments were conducted in compliance with protocols approved by the Institutional Animal Care and Use Committee of Southern Medical University (Approval Number: laec-2023-127). Post-acclimatization for one-week, the destabilization of the medial meniscus (DMM) surgery was performed to induce OA model under 2.5% isoflurane anesthesia using aseptic microsurgical techniques (Leica M80 stereomicroscope). The medial meniscal-tibial ligament in the right knee joint was selectively transected via medial parapatellar approach, with surgical success confirmed by > 1 mm meniscal subluxation upon passive joint manipulation. This procedure, which induces joint instability and closely mimics OA progression, is widely recognized as a reproducible model for studying OA pathology. Sham-operated mice received capsulotomy without ligament disruption.

One week post-surgery, the indicated adeno-associated viruses (aav, 1 × 10^11^ PFU/mL, 10 µL) was injected into the medial paratellar joint of corresponding mice using 33G Hamilton syringe under a micromanipulator. After a 7-week treatment period, knee tissues were collected for histopathological and molecular analyses. The aav-shNC (negative control), aav-shKDM1B, and aav-shSOX9 were obtained from Aiji Biotechnology (Guangzhou, Guangdong, China). The infection efficiency of the aav in mouse knee cartilage tissues was evaluated using RT-qPCR, western blot, or immunohistochemistry.

### Histopathological analysis

Freshly harvested knee joint samples were promptly immersion-fixed in 4% paraformaldehyde overnight and subsequently subjected to decalcification in 0.5 M EDTA with weekly solution renewal for two weeks. After undergoing alcohol gradient dehydration, the tissues were embedded in paraffin and sectioned. The sections were dewaxed in xylene, rehydrated through a graded alcohol series, and stained with hematoxylin and eosin (HE) and Toluidine blue (TB) for histological evaluation. Digital histomorphometry was conducted using an orthotopic light microscope (Leica, Cat. DMI1).

Osteochondral degeneration was stratified utilizing the Osteoarthritis Research Society International (OARSI) grading system, which is a standardized and widely accepted method for evaluating OA severity. This system quantifies structural changes in cartilage, including surface integrity, proteoglycan loss, and chondrocyte distribution, with higher scores indicating more advanced OA pathology. Synovial inflammation was evaluated using HE-stained synovial tissue sections, with a focus on inflammatory cell infiltration, synovial hyperplasia, and fibrosis. Severity of synovitis was scored on a scale of 0 to 9, with higher scores reflecting more pronounced inflammation. These standardized scoring systems ensure reliable and reproducible assessment of OA-related histopathological changes, consistent with established methodologies in OA.

### Immunohistochemistry and immunofluorescence analysis

For immunohistochemistry analysis, the paraffin-embedded sections underwent deparaffinization, rehydration, and antigen retrieval. Subsequently, endogenous peroxidase activity was quenched by incubating the sections with 3% hydrogen peroxide for 15 min. Next, the sections were blocked with 5% BSA for 1 h at room temperature and incubated overnight at 4℃ with primary antibodies against KDM1B (Abcam, ab198080, 1:300) and SOX9 (Abcam, ab155279, 1:500). Following this, the sections were incubated with horseradish peroxidase (HRP)-conjugated secondary antibodies for 2 h at room temperature. Signal visualization was carried out using the DAB horseradish peroxidase kit (Beyotime, China).

For immunofluorescence analysis, the paraffin sections were deparaffinized, hydrated, and underwent antigen retrieval. After blocking with 5% BSA for 1 h at room temperature, the sections were incubated with primary antibodies for F4/80 (Proteintech, 27044-1-AP, 1:200) and iNOS (CST, 13120 S, 1:200) overnight at 4℃. The sections were then stained with secondary antibodies for 1 h at room temperature, followed by nuclei labeling with DAPI. Images were acquired and analyzed using an inverted fluorescence microscope (Mshot, MF52) and the Image J 1.46r software (U. S. National Institutes of Health, Bethesda, Maryland, USA), respectively. As a negative control, the nonimmune anti-IgG antibody (Cell Signaling Technology, 2729, 1:50) was substituted for the primary antibody.

### Luciferase reporter assay

To delineate the regulatory mechanism of KDM1B on SOX9 transcriptional activity, we systematically dissected the SOX9 promoter by generating a series of truncation mutants. The full-length and various truncated SOX9 promoter fragments were cloned into the pGL3-basic luciferase reporter vector, generating constructs named SOX9 pro.-full, SOX9 pro.-Del1, SOX9 pro.-Del2, and SOX9 pro.-Del3, respectively. The reporter plasmids were transfected into the indicated chondrocytes using standard transfection protocols. At 24 h post-transfection, the cells were harvested and lysed. The Firefly and Renilla luciferase activities were measured using a Dual-luciferase reporter assay system (Promega, E1910). The firefly luciferase signal was normalized to the Renilla luciferase signal as an internal control for transfection efficiency.

### Chromatin Immunoprecipitation (ChIP)-qPCR assay

To map the epigenetic regulatory landscape of SOX9, we performed ChIP-qPCR using a ChIP assay kit (Abcam, ab500) to assess KDM1B occupancy and histone modification (H3K4me2) enrichment at the SOX9 promoter. Briefly, cross-linked protein-DNA complexes were immunoprecipitated using anti-KDM1B (Abcam, ab198080, 1:50), anti-H3K4me2 (Abcam, ab8898, 1:100), and anti-IgG (Cell Signaling Technology, 2729, 1:50) served as a negative control. After immunoprecipitation, the cross-linked protein-DNA complexes were reversed to free the DNA, which was then purified for subsequent RT-qPCR analysis. Specific primer sequences targeting the SOX9 promoter region used in the ChIP-qPCR assay are provided in Supplementary Table 1.

### RNA immunoprecipitation-qPCR (RIP-qPCR) assay

To investigate the interaction between NAT10 and KDM1B mRNA in chondrocytes, RIP-qPCR was performed using the EZ-Magna RIP™ RNA-Binding Protein Immunoprecipitation Kit (Millipore). Briefly, chondrocytes were lysed in pre-chilled RIP lysis buffer containing 1×protease inhibitor cocktail (Roche) and 20 U/mL RNaseOUT (Invitrogen). The lysates were incubated overnight at 4 °C with magnetic beads conjugated to an anti-NAT10 antibody (Abcam, ab194297, 1:100) or an anti-IgG antibody (Abcam, ab6715, 1:50) as a negative control. The immunoprecipitated RNA-protein complexes were subsequently eluted in RIP elution buffer and digested with proteinase K (Thermo Fisher). TRIzol reagent (Takara, Japan) was employed for RNA extraction. Finally, the purified RNA was reverse-transcribed into cDNA, and the KDM1B mRNA levels were quantified by RT-qPCR.

### Acetylated RNA immunoprecipitation-qPCR (acRIP-qPCR) assay

To evaluate the ac4C modification level on KDM1B mRNA, we adapted the methylated RNA immunoprecipitation (MeRIP) protocol with critical modifications for ac4C detection. In brief, total RNA was extracted from cultured cells and tissue samples using TRIzol reagent (Takara, Japan), followed by ribosomal RNA (rRNA) depletion using the IAseq FastSelect-rRNA HMR kit (No. 334376) to enrich mRNA. The purified RNA was then fragmented into approximately 200-nucleotide (nt) fragments using an automatic ultrasonic crusher. Next, The fragmented RNA was immunoprecipitated with an anti-ac4C antibody (1:50, ab252215, Abcam) or an anti-IgG antibody (1:50, ab6715, Abcam) as a negative control for 4 h at 4℃, after which the antibody-conjugated RNA samples were incubated with pretreated Protein A/G magnetic beads for 1 h at 4℃. Following extensive washing, the immunoprecipitated RNA was purified and subjected to RT-qPCR analysis to quantify the levels of ac4C-modified KDM1B mRNA.

### mRNA stability analysis

To systematically evaluate NAT10-mediated regulation of KDM1B mRNA stability, we employed a transcriptional arrest assay coupled with kinetic decay modeling. Briefly, the indicated chondrocytes were treated with 5 µg/mL actinomycin D (MCE, HY-17559), a transcriptional inhibitor. At 0, 3, 6, and 12 h after actinomycin D treatment, the corresponding cells were collected and their total RNA was extracted using TRIzol reagent (Takara, Japan). and tKDM1B mRNA decay kinetics were quantified using RT-qPCR.

### Statistical analysis

Quantitative data are presented as mean accompanied by standard deviation (SD). Comparative analyses between two experimental groups were conducted through Student’s t-test, and multi-group comparisons employed one-way ANOVA. These statistical evaluations were executed using GraphPad Prism 6.0 (GraphPad Software Inc., La Jolla, CA, USA). Correlation assessments for gene expression relationships were performed through Spearman rank correlation coefficient analysis. To enhance methodological reliability, all experimental procedures incorporated at least three biologically independent replications under identical conditions. The threshold for statistical significance was established at *p* < 0.05.

## Results

### Inhibition of KDM1B, which is aberrantly upregulated in osteoarthritis (OA), attenuates IL-1β-induced chondrocyte damage and ameliorates OA progression

**Fig. 1:**
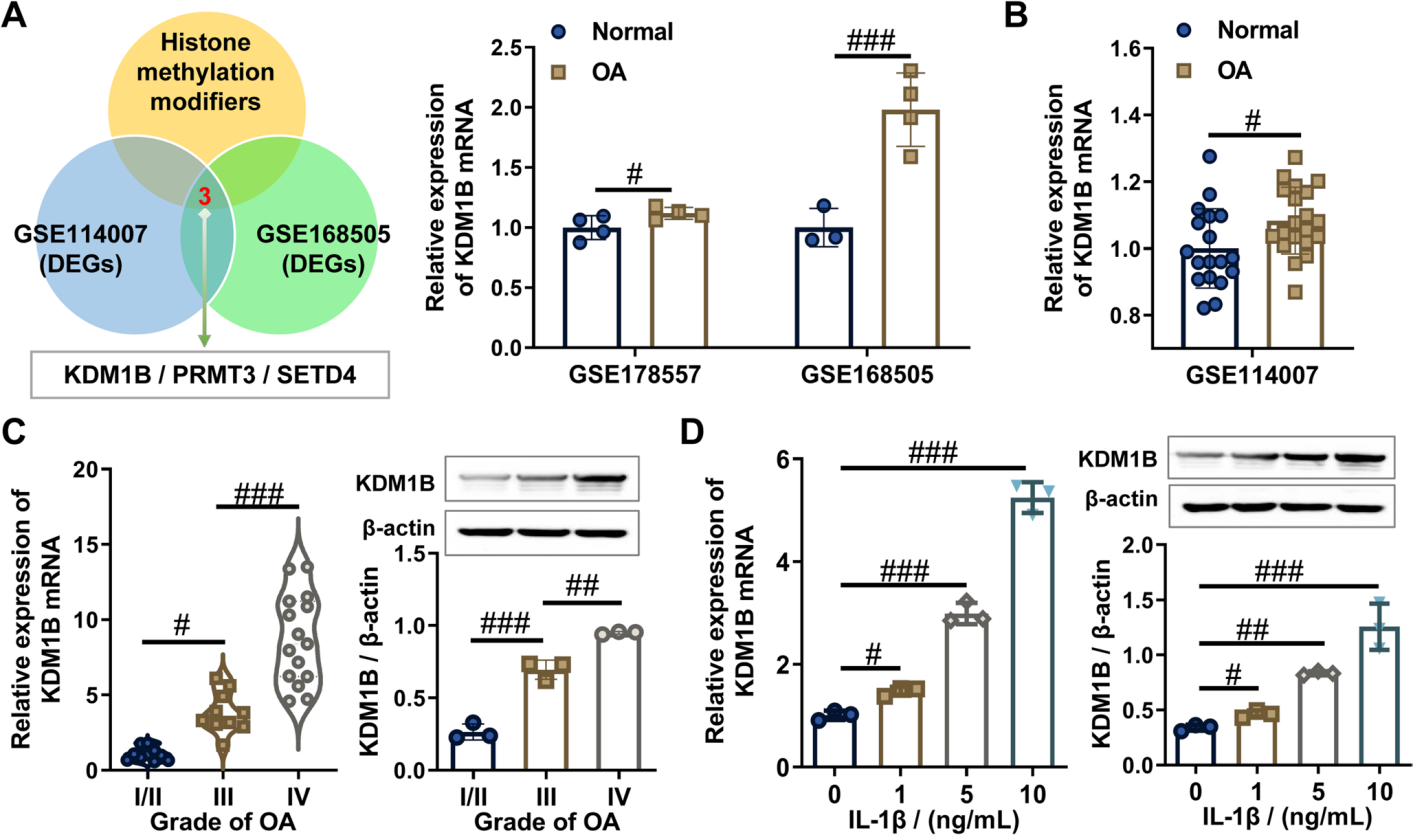
KDM1B expression was significantly upregulated in both IL-1β-stimulated chondrocytes and OA cartilage tissues, correlating positively with the severity of damage. **A** Venn diagram showed that differentially expressed histone methylation-modifying enzymes in normal and OA cartilage samples, and histogram showing KDM1B expression levels in 7 normal and 8 OA cartilage samples from GSE168505 and GSE178557 datasets. **B** Validation of KDM1B mRNA levels in 18 normal and 20 OA cartilage samples using GSE114007 dataset. **C** KDM1B mRNA and protein levels in OA cartilage at different stages were assessed by RT-qPCR and western blot. **D** KDM1B mRNA and protein levels in chondrocytes treated with IL-1β (0, 1, 5, and 10 ng/mL) were measured by RT-qPCR and western blot. Normal, non-OA cartilage; OA, osteoarthritis cartilage. *N* = 3, ^n.s^.*p* > 0.05, ^#^*p* < 0.05, ^##^*p* < 0.01, and ^###^*p* < 0.001

Through multi-omics interrogation of publicly accessible GEO repositories (GSE178557 and GSE168505), we systematically mapped the epigenetic landscape underlying osteoarthritic cartilage degeneration. The results revealed that three histone methylation modification enzymes (KDM1B, PRMT3, and SETD4) were aberrantly expressed in human OA cartilage tissues. Among these, KDM1B exhibited significant upregulation in OA cartilage tissues across both datasets, whereas PRMT3 and SETD4 showed inconsistent expression patterns (Fig. 1A, Supplementary Fig. 1A and B). Further validation using the GSE114007 dataset confirmed that only KDM1B was significantly upregulated in human OA cartilage tissues compared to normal tissues (Fig. 1B), whereas PRMT3 and SETD4 expression levels remained unchanged (Supplementary Fig. 1C). Strikingly, analysis of cartilage tissues from OA patients across various disease stages demonstrated that KDM1B expression both at the mRNA and protein levels progressively increased with OA progression (Fig. 1C). Consistent with these findings, KDM1B expression exhibited significant dose-dependent upregulation in IL-1β-induced chondrocytes, a widely recognized in vitro model of OA cartilage injury, as confirmed by RT-qPCR and western blot analysis (Fig. 1D). Collectively, this finding aligns with the GEO dataset analysis and clinical sample validation, highlighting the critical role of the histone demethylase KDM1B in OA pathogenesis due to its pronounced upregulation in OA cartilage tissues and chondrocytes.

**Fig. 2:**
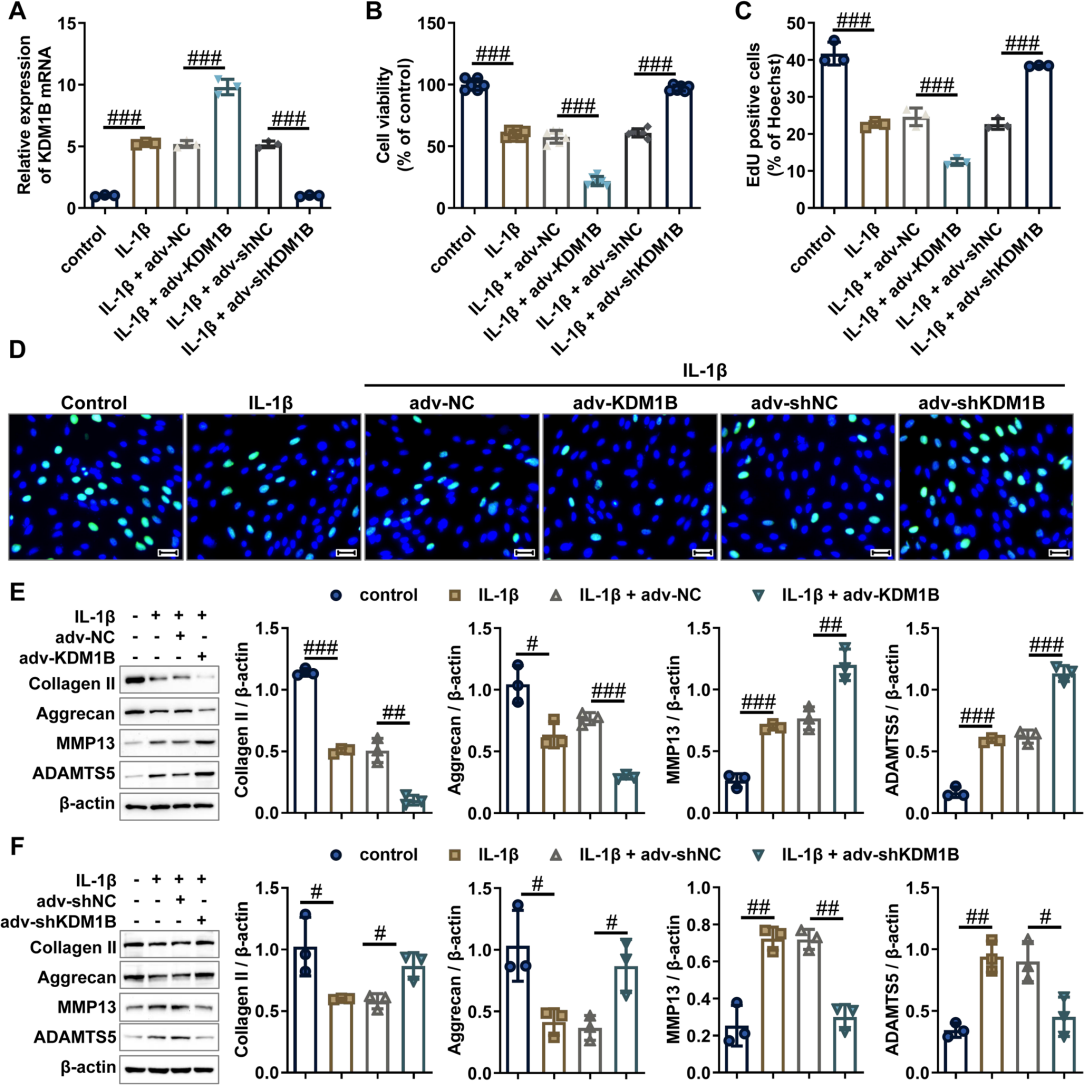
KDM1B catalyzes IL-1β-induced osteoarthritic chondrocyte phenotype. **A **KDM1B mRNA expression levels in the indicated chondrocytes were detected by RT-qPCR. **B-D **Cell proliferation ability was assessed by CCK-8 and EdU assays in the indicated chondrocytes. Scale bar = 50 μm. **E-F **The protein expression levels of ECM metabolism markers (MMP-13, ADAMTS5, Collagen II, and Aggrecan) were evaluated by western blot in the indicated chondrocytes. adv-NC, negative control adenovirus; adv-KDM1B, KDM1B-overexpressing adenovirus; adv-shNC, negative control short hairpin RNA adenovirus; adv-shKDM1B, KDM1B short hairpin RNA adenovirus. *N* = 3 ~ 6, ^#^*p* < 0.05, ^##^*p* < 0.01, and ^###^*p* < 0.001

Next, functional interrogation of KDM1B in chondrocyte pathobiology was conducted through gain- and loss-of-function approaches under IL-1β stimulation (10 ng/mL, 24 h). Western blot and RT-qPCR analyses revealed that shRNA-mediated KDM1B knockdown significantly suppressed IL-1β-induced KDM1B expression at both mRNA and protein levels, whereas KDM1B overexpression intensified this effect (Fig. 2A, Supplementary Figs. 2 and 3). Cellular proliferation dynamics assessed through CCK-8 and EdU assays demonstrated that KDM1B overexpression exacerbated IL-1β-induced inhibition of chondrocyte proliferation, whereas KDM1B knockdown alleviated this suppression (Fig. 2B-D). Consistent with these findings, overexpression of KDM1B significantly reduced the protein expression of the proliferation marker Ki-67 while increasing the expression of cell cycle inhibitors p27 and p21 in IL-1β-treated chondrocytes (Supplementary Fig. 4A), effects that were reversed in KDM1B knockdown (Supplementary Fig. 4B), suggesting that KDM1B modulates IL-1β-induced proliferative effects. Finally, ECM homeostasis analysis uncovered that overexpression of KDM1B significantly suppressed the protein expression levels of ECM anabolic markers (Collagen II and Aggrecan) as well as remarkably increased the protein expression levels of ECM catabolic enzymes (MMP13 and ADAMTS5) in IL-1β-induced chondrocytes (Fig. 2E), revealing that overexpression of KDM1B intensifies IL-1β-induced ECM metabolic imbalance in chondrocytes. Conversely, the suppression of KDM1B in IL-1β-stimulated chondrocytes triggered coordinated upregulation of ECM anabolic markers (Collagen II and Aggrecan) paired with effective downregulation of ECM catabolic enzymes (MMP13 and ADAMTS5), as quantified by western blot (Fig. 2F), indicating that KDM1B knockdown alleviates IL-1β-driven metabolic dysregulation in chondrocytes. Cumulative evidence establishes KDM1B as a pathogenic amplifier in osteoarthritic chondrocytes, where it drives IL-1β-induced damage in osteoarthritic chondrocytes through proliferative suppression with matrix homeostatic disruption. Targeted inhibition of KDM1B expression reverses these degenerative phenotypes, suggesting that it may be a viable molecular target for cartilage-protective therapies.

**Fig. 3:**
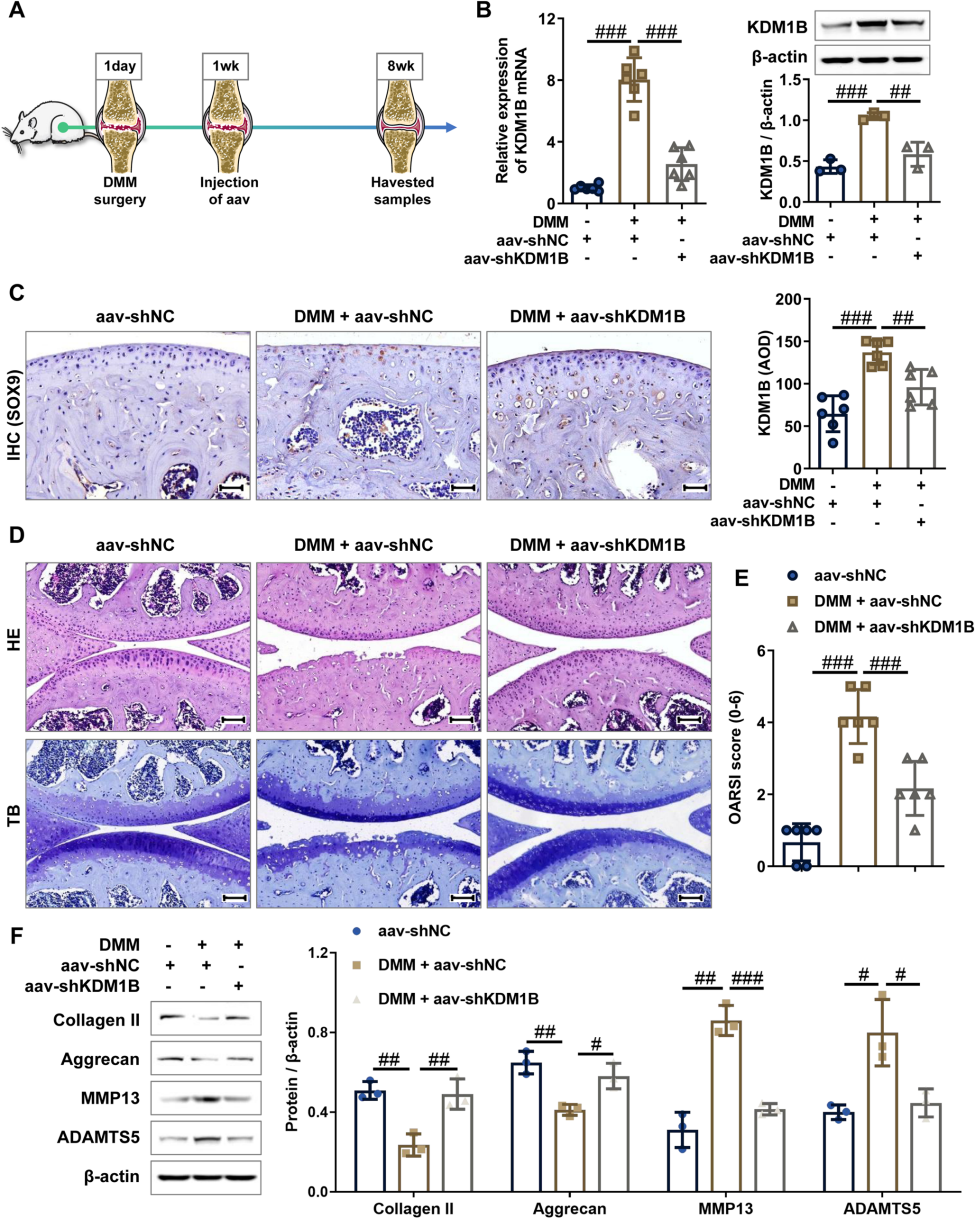
The aav-shKDM1B treatment alleviates OA progression in mice. **A** Schematic diagram of aav-shKDM1B for the treatment of DMM-induced OA mice. **B** The mRNA and protein levels of KDM1B in cartilage tissue from the indicated mice were evaluated by RT-qPCR and western blot. **C** Representative immunohistochemistry images and quantitative analysis of KDM1B expression in articular cartilage sections from experimental cohorts. Scale bars = 50 μm. **D** Representative hematoxylin and eosin (HE) staining and Toluidine blue (TB) staining of the knee joint sections from experimental cohorts. Scale bars = 100 μm. **E** OARSI scores was evaluated in cartilage tissue from the indicated mice. **F** Western blot analysis of ECM metabolic markers (MMP-13, ADAMTS5, Collagen II, and Aggrecan) in cartilage tissue from designated mice. DMM, destabilizing the medial meniscus-induced OA model; aav-shNC, negative control short hairpin RNA adeno-associated virus; aav-shKDM1B, KDM1B short hairpin RNA adeno-associated virus. AOD, average optical density. *N* = 3 ~ 6, ^#^*p* < 0.05, ^##^*p* < 0.01, and ^###^*p* < 0.001

Finally, we assessed the in vivo therapeutic effect of KDM1B inhibition by intra-articular injection of aav-shKDM1B or control aav-shNC into DMM-induced OA model mice (Fig. 3A). Multimodal validation confirmed effective KDM1B silencing in the cartilage tissues from OA mice, with significantly reduced mRNA and protein expression levels, as demonstrated by RT-qPCR, western blot, and immunohistochemistry (Fig. 3B and C). Histomorphometric evaluation through HE and TB staining revealed pronounced cartilage structural abnormalities in OA mice, characterized by surface fibrillation, proteoglycan depletion, and chondrocyte disorganization, and these pathological alterations were substantially reversed post-aav-shKDM1B treatment (Fig. 3D). The therapeutic effect of avv-shKDM1B was further confirmed by OARSI scoring system, which showed that knockdown of KDM1B significantly attenuated the cartilage degeneration process in OA mice (Fig. 3E). Consistent with in vitro findings, western blot analysis demonstrated that KDM1B knockdown significantly upregulated the expression of ECM anabolic markers (Collagen II and Aggrecan) and downregulated the expression of ECM catabolic markers (MMP-13 and ADAMTS5) in the cartilage tissues from OA mice (Fig. 3F), further supporting the protective role of KDM1B knockdown against articular cartilage degeneration. Additionally, we evaluated the impact of aav-shKDM1B on synovitis in OA mice. Histopathological evaluation through HE staining demonstrated significant attenuation in synovial tissue pathology after aav-shKDM1B intervention, with observable decreases in both synovial hyperplasia and inflammatory cell infiltration (Supplementary Fig. 5A). Consistent with these morphological improvements, quantitative analysis with synovitis scores also showed that aav-shKDM1B treatment could outstandingly reduce the synovitis scores of OA mice (Supplementary Fig. 5B), further suggesting that targeted KDM1B suppression through aav-shKDM1B administration effectively alleviates synovitis in OA mice. To explore the underlying mechanisms, immunofluorescence analysis was performed to assess macrophage polarization in synovial tissues. In OA mice, the percentage of F4/80-positive cells and iNOS-positive cells was significantly increased but markedly reduced following aav-shKDM1B treatment (Supplementary Fig. 5C and D), suggesting that aav-shKDM1B ameliorates synovitis partly by inhibiting M1 macrophage polarization. In a nutshell, the above data demonstrate that intra-articular delivery of aav-shKDM1B attenuates OA progression by reducing cartilage degradation and synovitis, underscoring KDM1B as a therapeutic target for OA treatment.

### NAT10-mediated ac4C modification enhances KDM1B mRNA stability and drives its pathological upregulation in osteoarthritis

**Fig. 4:**
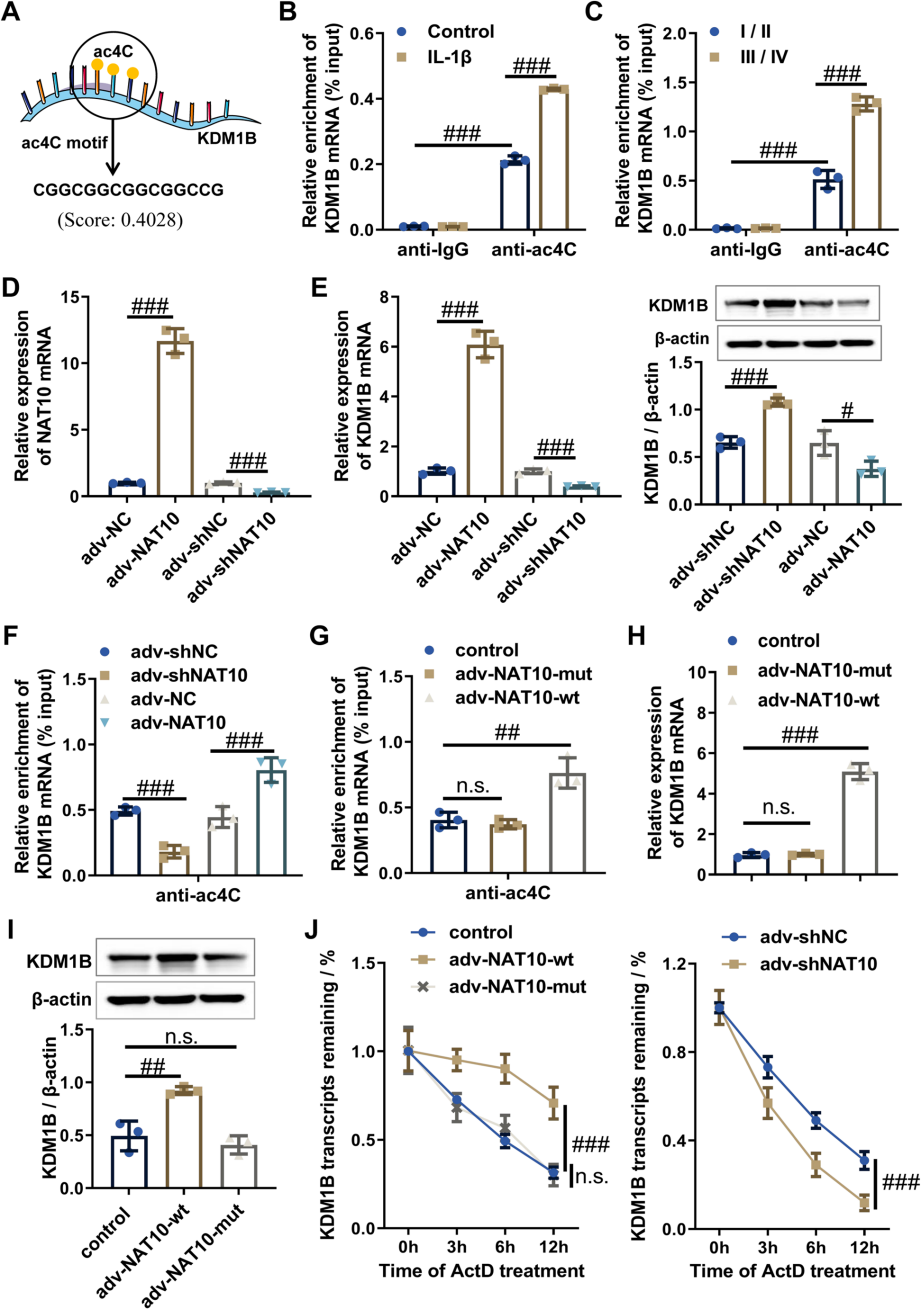
Consistent upregulation of KDM1B in both IL-1β-stimulated chondrocytes and OA cartilage tissues is driven by NAT10/ac4C-dependent mRNA stabilization. **A** PACES prediction identifying ac4C modification site on KDM1B mRNA (position: 23–37; Score: 0.4028). **B** acRIP-qPCR analysis was performed to confirm the ac4C modification levels of KDM1B mRNA in chondrocytes with or without **IL-1β**. **C** acRIP-qPCR analysis was performed to confirm the ac4C modification levels of KDM1B mRNA in cartilage tissues of different OA pathological grades. **D** The mRNA expression levels of NAT10 were measured by RT-qPCR in chondrocytes transfected with adv-NAT10 or adv-shNAT10. **E** The mRNA and protein expression levels of KDM1B were measured by RT-qPCR and western blot in chondrocytes with NAT10 overexpression or knockdown. **F** acRIP-qPCR assessing the ac4C modification levels of KDM1B mRNA in chondrocytes with NAT10 overexpression or knockdown. **G** acRIP-qPCR analysis confirming the ac4C modification levels of KDM1B mRNA in chondrocytes with NAT10-wt or NAT10-mut overexpression. **H** The mRNA expression of KDM1B was measured by RT-qPCR in the chondrocytes with NAT10-wt or NAT10-mut overexpression. **I** The protein expression of KDM1B was measured by western blot in the chondrocytes with NAT10-wt or NAT10-mut overexpression. **J** The indicated chondrocytes were treated with 5 µg/mL actinomycin D for 0, 3, 6, and 12 h, the mRNA expression of KDM1B was measured by RT-qPCR. Normal, non-OA cartilage; OA, osteoarthritis cartilage; adv-NC, negative control adenovirus; adv-KDM1B, KDM1B-overexpressing adenovirus; adv-shNC, negative control short hairpin RNA adenovirus; adv-shKDM1B, KDM1B short hairpin RNA adenovirus. *N* = 3, ^n.s^.*p* > 0.05, ^##^*p* < 0.01, and ^###^*p* < 0.001

Bioinformatics analysis using the PACES algorithm (http://www.rnanut.net/paces/) identified a conserved ac4C modification motif within the KDM1B transcript (Fig. 4A). To functionally characterize these motifs, we implemented acRIP-qPCR in human chondrocytes with or without IL-1βstimulation. The acRIP-qPCR results demonstrated significant enrichment of KDM1B in anti-ac4C antibody precipitates compared to anti-IgG antibody, with this enrichment being significantly augmented in IL-1β-treated chondrocytes (Fig. 4B). In line with the in vitro cell model findings, we also observed significant enrichment of KDM1B in anti-ac4C immunoprecipitates from OA cartilage tissue, and this enrichment was significantly greater in grade III/IV OA cartilage compared to grade I/II OA tissue (Fig. 4C). These findings indicate that KDM1B mRNA undergoes aberrant ac4C modification in OA may be a key factor contributing to the dysregulation of KDM1B in OA pathogenesis.

As the exclusive mammalian mRNA acetyltransferase mediating ac4C deposition [19], we conducted gain- and loss-of-function experiments to investigate NAT10’s regulatory role in chondrocytes. RT-qPCR and western blot results showed that NAT10 overexpression significantly increased KDM1B expression at both mRNA and protein levels, while NAT10 knockdown resulted in decreased KDM1B expression (Fig. 4D and E**)**. Meanwhile, acRIP-qPCR further confirmed that KDM1B mRNA enrichment with anti-ac4C antibody was significantly higher in NAT10-overexpressing chondrocytes but reduced in NAT10-knockdown chondrocytes (Fig. 4F). To investigate the role of NAT10’s acetyltransferase activity, we constructed adenoviruses expressing wild-type NAT10 (aav-NAT10-wt) and catalytically inactive G641E mutant (adv-NAT10-mut) [20]. The acRIP-qPCR results demonstrated that NAT10-wt overexpression increased KDM1B mRNA enrichment with anti-ac4C antibody, whereas NAT10-mut did not (Supplementary Fig. 6A, Fig. 4G). Consistently, NAT10-wt but not NAT10-mut significantly upregulated KDM1B expression at both mRNA and protein levels (Fig. 4H and I), indicating that NAT10 regulates KDM1B expression primarily through its RNA acetylation activity. Subsequently, mRNA stability assays revealed that NAT10 knockdown resulted in a noticeable decrease the KDM1B mRNA stability, whereas overexpression of NAT10-wt but not NAT10-mut significantly increased the KDM1B mRNA stability (Fig. 4J), confirming that NAT10 maintains KDM1B mRNA stability in an ac4C-dependent manner. Additionally, NAT10 knockdown in IL-1β-induced chondrocytes effectively attenuated KDM1B levels (Supplementary Fig. 6B and C) and restored extracellular matrix (ECM) metabolism disorders, evidenced by Collagen II, Aggrecan, MMP13, and ADAMTS5 normalization (Supplementary Fig. 6D and E), suggesting that NAT10 modulation as a disease-modifying intervention for cartilage maintenance. In a nutshell, the consolidated data delineate an ac4C-dependent stabilization mechanism wherein NAT10-mediated mRNA acetylation increases KDM1B mRNA stability, thereby driving its pathological accumulation in OA cartilage tissues and chondrocytes.

### KDM1B directly binds to the SOX9 promoter and catalyzes H3K4me2 demethylation to suppress SOX9 expression

**Fig. 5:**
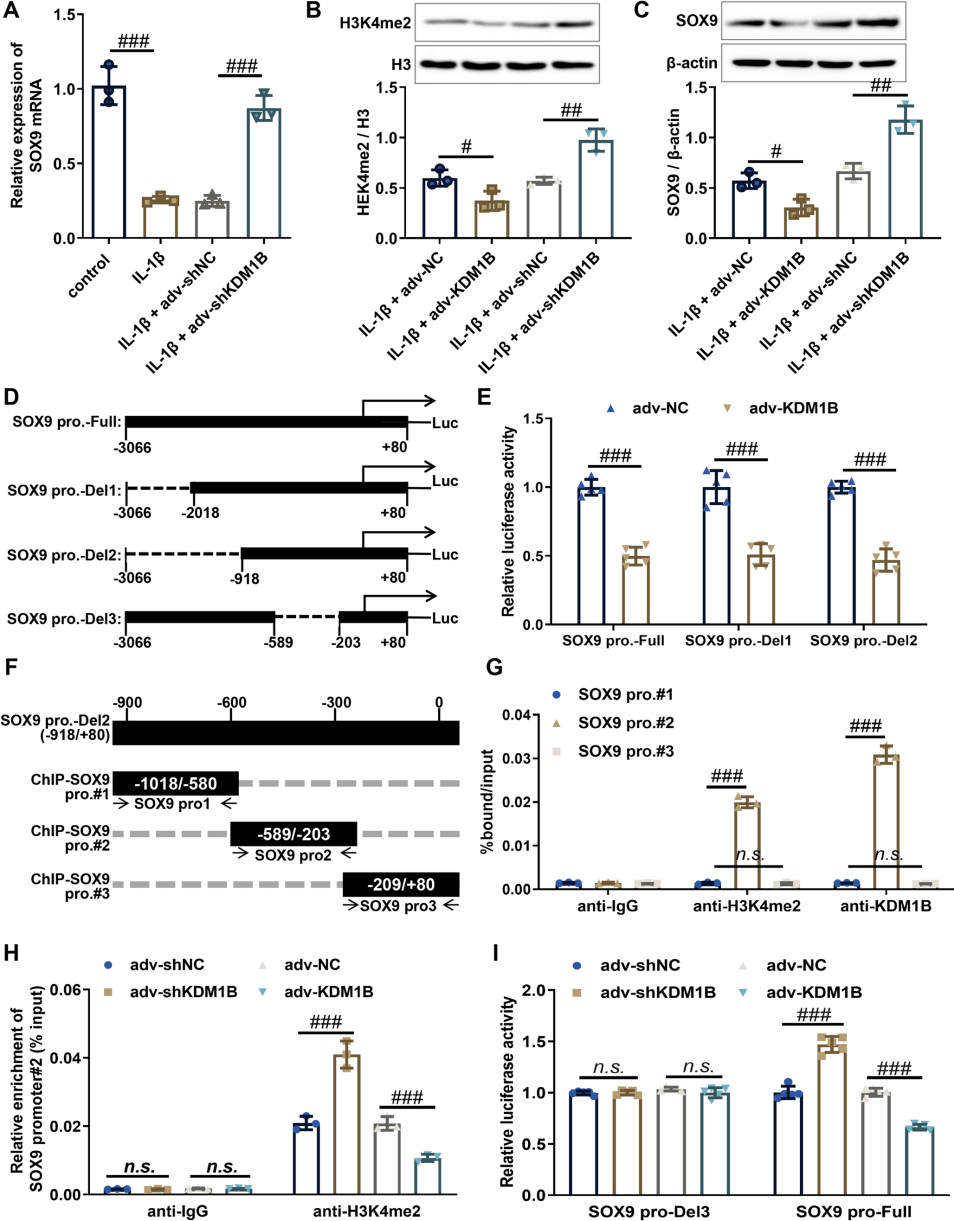
KDM1B directly binds to the SOX9 promoter and catalyzes H3K4me2 demethylation to suppress SOX9 expression. **A** SOX9 mRNA expression levels in the indicated chondrocytes were detected by RT-qPCR. **B** Western blot was used to measure the protein expression levels of H3K4m2 in the indicated chondrocytes. **C** Western blot was used to measure SOX9 protein levels in the indicated chondrocytes. **D** Construction of SOX9 promoter truncated mutation reporter vectors (SOX9 pro.-Full, SOX9 pro.-Del1, SOX9 pro.-Del2, and SOX9 pro.-Del3). **E** Luciferase reporter assays were performed in KDM1B overexpressing chondrocytes transfected with SOX9 pro.-Full, SOX9 pro.-Del1, or SOX9 pro.-Del2. **F** The positions of three pairs of primers completely covering the Del2 region of SOX9 promoter were designed. **G **ChIP-qPCR using anti-KDM1B and anti-H3K4me2 antibodies analyzed KDM1B and H3K4me2 enrichment in this region. **H** ChIP-qPCR was used to analyze the effect of KDM1B overexpression or knockdown on H3K4me2 level in SOX9 pro.#2. **I** Luciferase assays were performed in chondrocytes following KDM1B overexpression or knockdown, co-transfected with SOX9 pro.-Del3 or SOX9 pro.-Full. SOX9 pro., SOX9 promoter; adv-NC, negative control adenovirus; adv-KDM1B, KDM1B-overexpressing adenovirus; adv-shNC, negative control short hairpin RNA adenovirus; adv-shKDM1B, KDM1B short hairpin RNA adenovirus; DMM, destabilizing the medial meniscus-induced OA mice; aav-shNC, negative control short hairpin RNA adeno-associated virus; aav-shKDM1B, KDM1B short hairpin RNA adeno-associated virus. *N* = 3, ^n.s^.*p* > 0.05, ^#^*p* < 0.05, ^##^*p* < 0.01, and ^###^*p* < 0.001

Emerging evidence highlights that SRY-box transcription factor 9 (SOX9) plays a protective role in mitigating cartilage degeneration in OA [5, 21, 22]. Analysis of GSE114007 repository revealed that SOX9 mRNA levels were significantly lower in OA cartilage tissues compared to normal tissues, with a strong negative correlation between KDM1B and SOX9 expression (Supplementary Fig. 7A). Advanced-stage OA cartilage tissues (grade III/IV) exhibited lower SOX9 mRNA levels than early-stage tissues (grade I/II), and KDM1B levels inversely correlated with SOX9 expression in human cartilage tissues (Supplementary Fig. 7B). In vitro and in vivo experiments demonstrated that KDM1B knockdown increased SOX9 mRNA expression in IL-1β-treated chondrocytes and in the cartilage of DMM-induced OA mice (Fig. 5A, Supplementary Fig. 7C). These findings suggest that KDM1B negatively regulates SOX9 expression, implicating SOX9 as a key mediator of KDM1B-driven OA progression.

In line with prior evidence that the SOX9 promoter region is significantly enriched with H3K4me2 [23, 24], our study further elucidated the role of KDM1B in regulating SOX9 expression through H3K4me2 demethylation. In IL-1β-treated chondrocytes, KDM1B knockdown significantly increased the protein levels of both H3K4me2 and SOX9, whereas ectopic KDM1B expression had the opposite effect (Fig. 5B and C). As expected, in vivo experiments also revealed that KDM1B suppression restored the protein expression of both H3K4me2 and SOX9 in the cartilage tissues of OA mice (Supplementary Fig. 7D), supporting KDM1B’s role as an epigenetic repressor. Therefore, we hypothesized that KDM1B could directly suppress SOX9 expression by catalyzing H3K4me2 demethylation at the SOX9 promoter.

To uncover direct transcriptional regulation, we performed luciferase reporter assays with different truncated SOX9 promoter constructs (SOX9 pro.-Full, SOX9 pro.-Del1, and SOX9 pro.-Del2) in chondrocytes (Fig. 5D). The results demonstrated that overexpression of KDM1B could significantly inhibited the luciferase activity of the SOX9 pro.-Full, SOX9 pro.-Del1, and SOX9 pro.-Del2 (Fig. 5E), indicating that the KDM1B binding site likely resides within the SOX9 pro.-Del2 region (−918/+80).

To pinpoint the exact binding region, we designed ChIP primers covering the SOX9 pro.-Del2 region (−918/+80) for quantitative analysis (Fig. 5F). ChIP-qPCR identified pronounced co-enrichment of KDM1B and H3K4me2 at the region amplified by ChIP-SOX9 pro.#2 primers (Fig. 5G), pinpointing this subregion as the interaction hub. Further ChIP-qPCR validation demonstrated that KDM1B overexpression significantly reduced H3K4me2 levels at the SOX9 pro.#2 region, whereas KDM1B knockdown increased H3K4me2 levels (Fig. 5H), confirming that KDM1B binds to the SOX9 promoter region (−589/−203) and catalyzes H3K4me2 demethylation to suppress SOX9 transcription. To further confirmed the necessity of this sequence for KDM1B-mediated transcriptional regulation, we constructed SOX9 pro.-Del3 luciferase reporter vector (SOX9 promoter − 568/−203 region deletion, Fig. 5C) to perform luciferase reporter assays. The results from luciferase reporter assays showed that KDM1B modulation robustly altered the luciferase activity of SOX9 pro.-Full in chondrocyte but had no effect on the luciferase activity of SOX9 pro.-Del3 (Fig. 5I). Ultimately, these data demonstrate that KDM1B directly binds to the SOX9 promoter at the − 589/−203 region, where it erases H3K4me2 marks to suppress SOX9 transcription.

### KDM1B drives osteoarthritic chondrocyte injury and cartilage degradation through transcriptional repression of SOX9

**Fig. 6:**
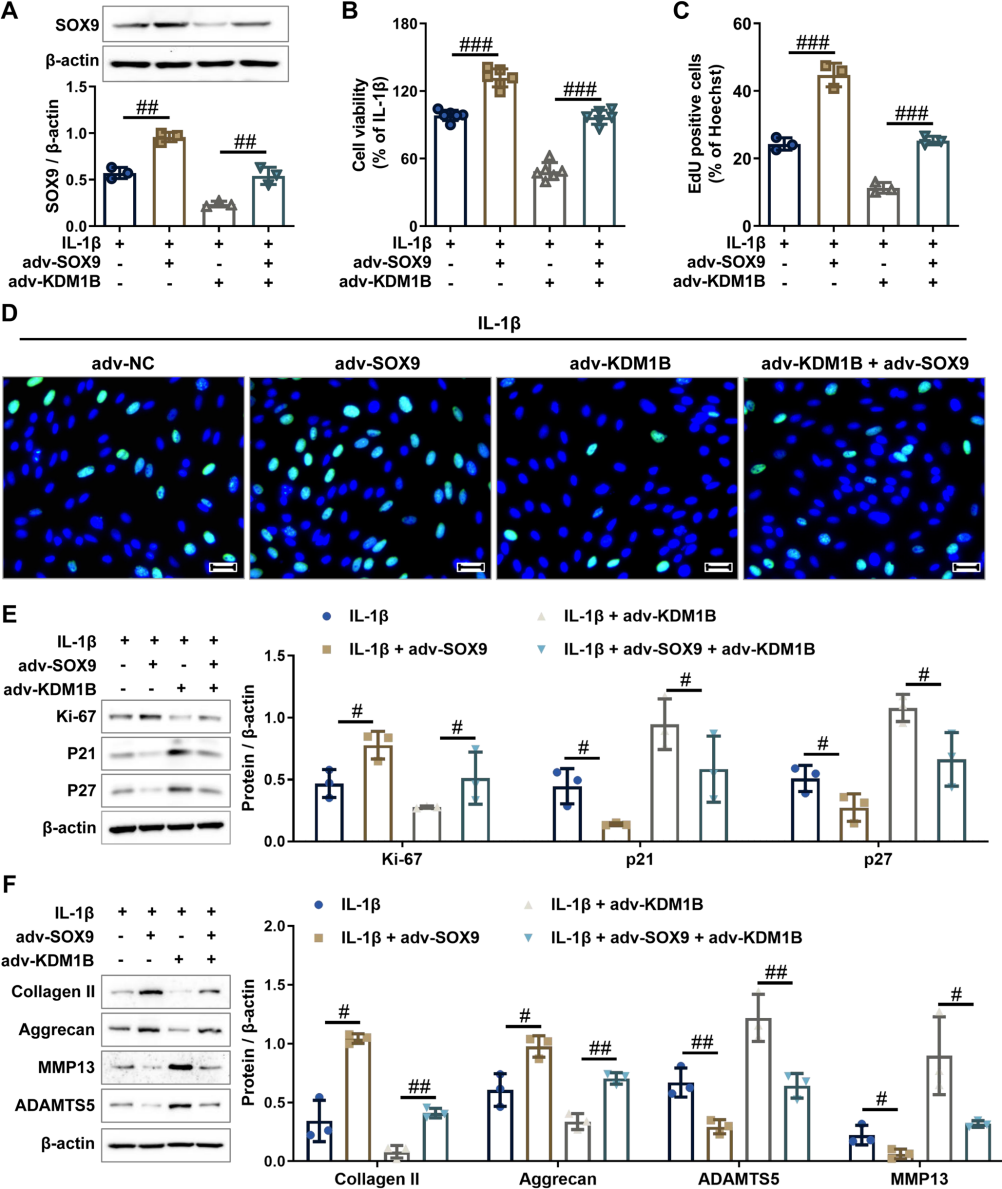
KDM1B exacerbates IL-1β-induced chondrocyte injury by suppressing SOX9 expression. **A.** The protein expression level of SOX9 in the indicated chondrocytes was assessed by western blot. **B-D.** The proliferation of the indicated chondrocytes was detected by CCK-8 and EdU assays. Scale bar = 50 μm. **E.** The protein expression levels of proliferation-related markers (Ki67, p21, and p27) were detected by western blot in the indicated chondrocytes. **F.** The protein expression levels of ECM metabolic markers (MMP13, ADAMTS5, Collagen II, and Aggrecan) were detected by western blot in the indicated chondrocytes. adv-NC, negative control adenovirus; adv-KDM1B, KDM1B-overexpressing adenovirus; adv-SOX9, SOX9-overexpressing adenovirus. *N* = 3 ~ 6, ^#^*p* < 0.05, ^##^*p* < 0.01, and ^###^*p* < 0.001

To determine whether KDM1B promotes osteoarthritic chondrocyte injury in a SOX9-dependent manner, rescue studies were conducted in interleukin-1β-stimulated chondrocytes. In IL-1β-treated chondrocytes, adv-SOX9 treatment effectively restored SOX9 levels that were diminished by adv-KDM1B treatment, with both vectors administration normalizing expression patterns (Fig. 6A). Cellular function analysis by CCK-8 and EdU assays revealed that adv-SOX9 or adv-KDM1B single interventions significantly mitigated or amplified IL-1β-induced growth suppression, while combined treatment neutralized these effects (Fig. 6B and D). Western blot analysis further confirmed that adv-SOX9 or adv-KDM1B single interventions increased or decreased the protein expression of Ki-67 along with decreased or increased the protein expression of p27 and p21 in IL-1β-induced chondrocytes, and the above phenomena was abrogated by co-interventions with adv-SOX9 and adv-KDM1B (Fig. 6E), confirming that KDM1B exacerbates IL-1β-induced chondrocyte proliferation inhibition by SOX9 downregulation. In addition, western blot results also showed that adv-SOX9 treatment triggered coordinated upregulation of ECM anabolic markers (Collagen II and Aggrecan) paired with effective downregulation of ECM catabolic enzymes (MMP13 and ADAMTS5) in IL-1β-stimulated chondrocytes, whereas adv-KDM1B treatment intensified ECM anabolic markers (Collagen II and Aggrecan) protein inhibition and exacerbated ECM catabolic enzymes (MMP13 and ADAMTS5) protein upregulation, and this function was significantly attenuated by co-treatment with adv-SOX9 and adv-KDM1B (Fig. 6F), suggesting that KDM1B aggravates IL-1β-induced ECM metabolic imbalance through SOX9 inhibition. In summary, these comprehensive findings demonstrate that KDM1B drives chondrocyte impairment and matrix degradation through transcriptional repression of SOX9, revealing this molecular axis as a promising target for therapeutic intervention in OA cartilage injury.

**Fig. 7:**
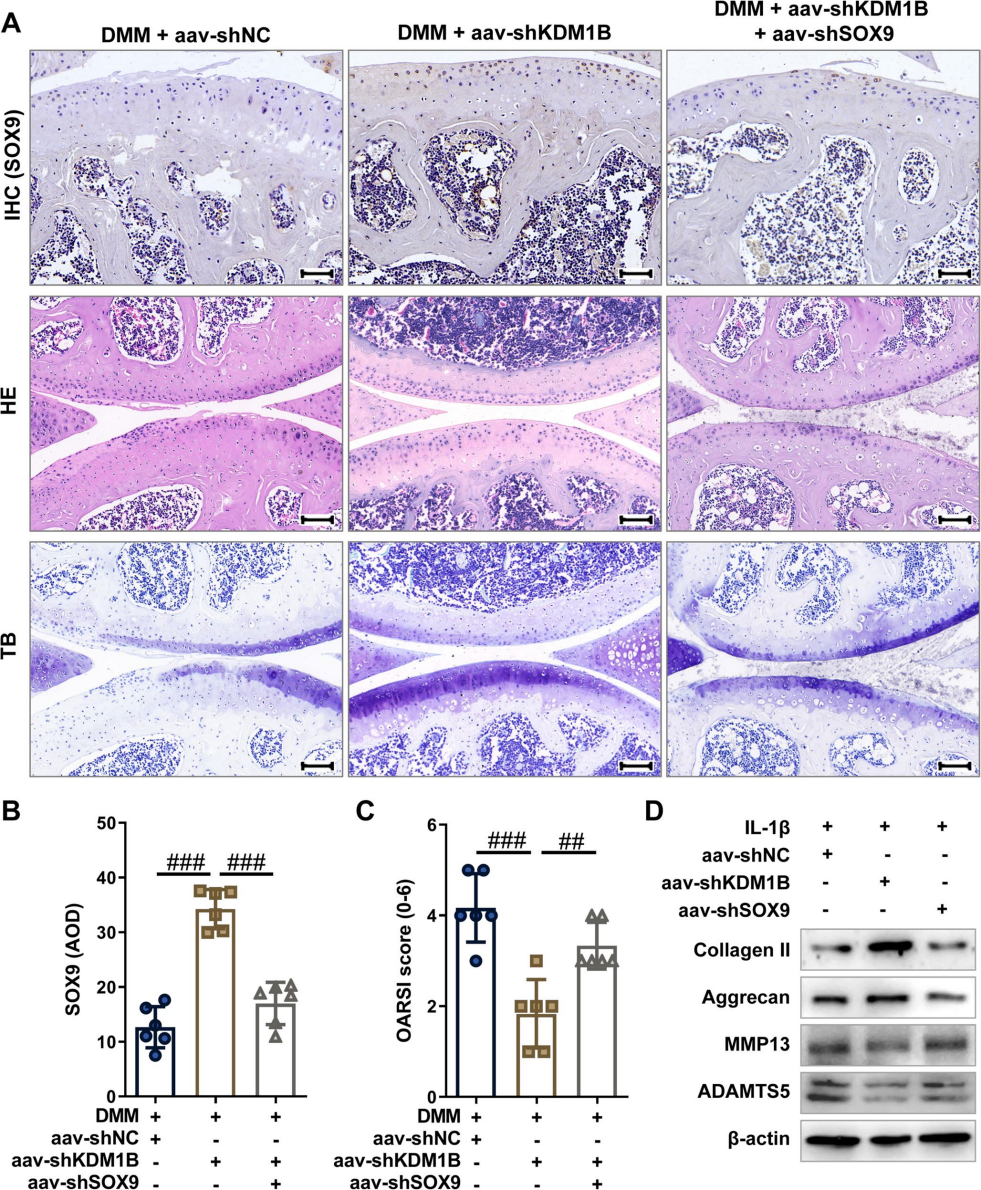
KDM1B knockdown alleviates OA development through enhancing SOX9 expression. **A** Representative immunohistochemistry (SOX9), hematoxylin-eosin (HE), and toluidine blue (TB) staining images of knee joint articular cartilage sections from experimental cohorts. Scale bars = 50 μm (IHC)/100 μm (HE, TB). **B** Quantitative histomorphometric analysis of SOX9-positive cell density in articular cartilage across experimental groups. **C** OARSI scores was evaluated in cartilage tissue from the indicated mice. **D** The protein expression levels of ECM metabolic markers (MMP-13, ADAMTS5, Collagen II, and Aggrecan) in the indicated cartilage tissue were evaluated by western blot. DMM, destabilizing the medial meniscus-induced OA mice; aav-shNC, negative control short hairpin RNA adeno-associated virus; aav-shKDM1B, KDM1B short hairpin RNA adeno-associated virus; aav-shSOX9, SOX9 short hairpin RNA adeno-associated virus. AOD, average optical density. *N* = 3 ~ 6, ^##^*p* < 0.01, and ^###^*p* < 0.001

Building on mechanistic insights from cellular studies, complementary rescue experiments were conducted in DMM - induced OA mice. Immunohistochemical analysis revealed that aav-shKDM1B elevated SOX9 levels in articular cartilage, an effect neutralized through concurrent aav-shSOX9 treatment (Fig. 7A and B). The results from HE and TB staining demonstrated that aav-shKDM1B reduced structural degeneration and improved OARSI scores, benefits abolished by SOX9 suppression (Fig. 7A and C), suggesting that KDM1B knockdown mitigates cartilage damage in OA mice by upregulating SOX9 expression. Consistent with in vitro results, western blot analysis showed that aav-shSOX9 treatment reversed the effects of aav-shKDM1B on promoting the expression of anabolic markers (Collagen II and Aggrecan) and suppressing the expression of catabolic markers (MMP13 and ADAMTS5) in the cartilage tissues of OA mice (Fig. 7D), further confirming that SOX9-dependent ECM stabilization as a key mechanism of KDM1B targeting. Additionally, synovial pathology assessment further revealed that aav-shKDM1B attenuated synovial hyperplasia, inflammatory cell infiltration, and synovial scores, outcomes negated by SOX9 interference (Supplementary Fig. 8A and B). Immunofluorescence analysis revealed that aav-shSOX9 reversed restored synovial macrophage infiltration (F4/80^+^) and pro-inflammatory M1 polarization (iNOS^+^), both suppressed by KDM1B silencing (Supplementary Fig. 8C and D). Collectively, these in vivo data demonstrate that targeted KDM1B suppression mitigates cartilage degradation and synovial inflammation by restoring SOX9 expression, highlighting the KDM1B-SOX9 axis as a critical therapeutic target for OA.

### NAT10 depletion protects chondrocytes from osteoarthritic injury by modulating the KDM1B/SOX9 axis

**Fig. 8:**
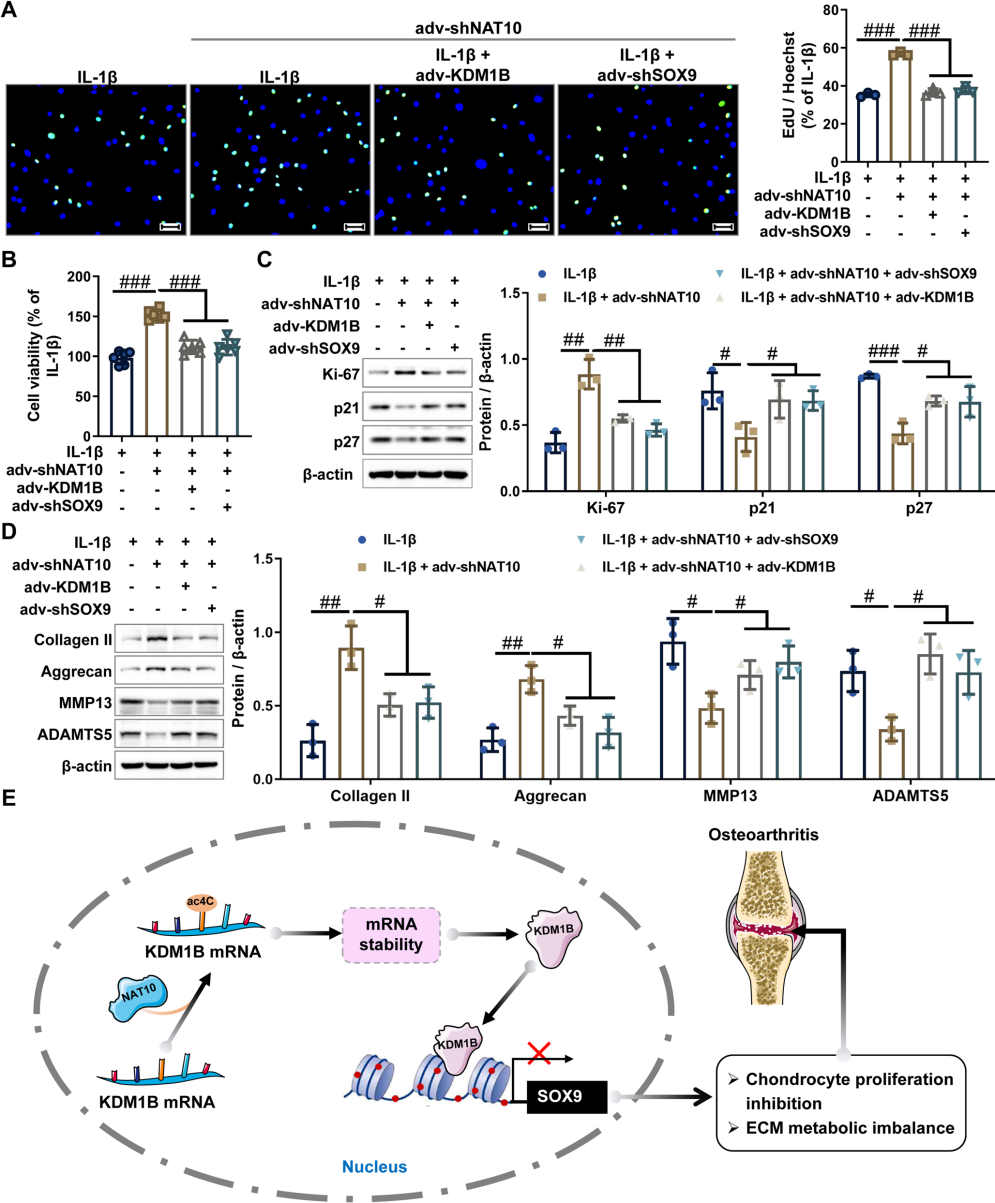
Knockdown of NAT10 can improve IL-1β-induced proliferation inhibition and ECM metabolism imbalance by regulating the KDM1B/SOX9 axis in chondrocytes. **A-B **The proliferation of the indicated chondrocytes was assessed by CCK-8 and EdU assays. Scale bar = 100 μm. **C.** The protein expression levels of proliferation-related markers (Ki67, p21, and p27) were detected by western blot in chondrocytes. **D **The protein expression levels of ECM metabolic markers (MMP13, ADAMTS5, Collagen II, and Aggrecan) were detected by western blot in chondrocytes. **E.** The schematic diagram summarizes the molecular mechanisms by which KDM1B promotes OA development, providing a comprehensive overview of the NAT10/ac4C/KDM1B/SOX9 pathway. adv-NC, negative control adenovirus; adv-KDM1B, KDM1B-overexpressing adenovirus; adv-shNC, negative control short hairpin RNA adenovirus; adv-shSOX9, SOX9 short hairpin RNA adenovirus; adv-shNAT10, NAT10 interfering adenovirus. *N* = 3 ~ 6, ^#^*p* < 0.05, ^##^*p* < 0.01, and ^###^*p* < 0.001

To elucidate NAT10T functions through the regulation of KDM1B/SOX9 axis, we conducted complementary rescue studies in an IL-1β-induced chondrocyte injury model. The results from CCK-8 and EdU assays demonstrated that knockdown of NAT10 abrogated IL-1β-driven proliferation inhibition in chondrocytes, this protective effect was nullified by either KDM1B upregulation or SOX9 suppression (Fig. 8A and B). Consistent with these findings, western blot analysis revealed that NAT10 knockdown upregulated Ki-67 protein levels and downregulated the protein levels of both p27 and p21 in IL-1β-treated chondrocytes, with these effects negated by KDM1B overexpression or SOX9 knockdown (Fig. 8C), further confirming that the KDM1B/SOX9 axis mediates the protective effects of NAT10 knockdown on chondrocyte proliferation. Additionally, NAT10 knockdown elevated anabolic markers (Collagen II and Aggrecan) protein levels and suppressed catabolic markers (MMP-13 and ADAMTS5) protein levels in IL-1β-treated chondrocytes, these regulatory effects were also erased by KDM1B overexpression or SOX9 knockdown (Fig. 8D), indicating that the correction of NAT10 knockdown on ECM metabolic imbalance is dependent on KDM1B/SOX9 axis. Taken together, these results demonstrate that NAT10 suppression promotes chondrocyte proliferation and maintains ECM homeostasis through epigenetic regulation of the KDM1B/SOX9 axis, thereby alleviating IL-1β-induced chondrocyte damage, and this pathway holds promise as a therapeutic target for OA.

## Discussion

The incomplete understanding of OA pathogenesis has impeded the development of effective therapies [25]. Therefore, identifying key molecules involved in OA pathogenesis is crucial for designing therapeutic targets and developing novel drugs. In this study, we demonstrated that KDM1B/SOX9 molecular axis as a key regulator of OA progression through comprehensive in vitro and in vivo studies. Mechanistically, NAT10-mediated ac4C modification enhances KDM1B mRNA stability, driving its abnormal upregulation in OA. The elevated KDM1B levels subsequently exerts pathogenic effects by binding to the SOX9 promoter region and catalyzing the demethylation of H3K4me2, thereby suppressing SOX9 expression. These findings not only uncover a novel molecular mechanism underlying OA pathogenesis but also highlight the NAT10/ac4C/KDM1B/SOX9 axis as a promising therapeutic target for OA.

Histone methylation is a dynamic epigenetic modification that modulates gene transcription by histone methyltransferases (HMTs) and histone demethylases (HDMTs) [26]. According to their sequence homology and catalytic mechanism, HMTs are categorized into KDM1 family and Jumonji C(JmjC) KDM family. The KDM1 family, which includes KDM1A and KDM1B (a homology of KDM1A with less than 31% similarity), plays vital roles in regulating epigenetic modifications in both physiological and pathological conditions [27, 28]. Accumulating evidence has elucidated the role of KDM1A in chondrocytes, positioning it as a potential therapeutic target for OA and other arthritic conditions as a potential target for the treatment of OA and other arthritis. For example, KDM1A-mediated H3K9 demethylation contributes to IL-1β-induced microsomal prostaglandin E synthase 1 expression, thereby driving OA progression [29]. Additionally, KDM1A suppresses articular chondrocyte anabolism, exacerbating OA progression [30]. However, the function of KDM1B in OA remained unexplored until this investigation. Our study demonstrates that KDM1B is significantly upregulated in OA cartilage tissues and chondrocytes, and its overexpression worsens IL-1β-induced chondrocyte dysfunction. Conversely, KDM1B knockdown ameliorates OA progression by enhancing chondrocyte proliferation and restoring ECM homeostasis. These findings identify KDM1B as a novel contributor to OA pathogenesis and highlight its therapeutic target.

Building on these observations, we further investigated the regulatory mechanisms of KDM1B-driven cartilage degeneration in OA, particularly focusing on SOX9. As a pivotal chondroprotective transcription factor, SOX9 maintains articular cartilage integrity through mechanisms potentially influenced by epigenetic modifications [21, 31–33]. For instance, Kim KI et al. reported that altered histone methylation patterns, particularly at H3K9 and H3K27 sites in OA chondrocytes, correlate with SOX9 downregulation [33]. Zhang et al. demonstrated that age-related depletion of H3K4me2 at the SOX9 promoter has been linked to reduced transcriptional activity, with KDM1A deficiency shown to preserve H3K4me2 levels and enhance SOX9 expression in adult mouse chondrocytes [24]. These findings prompted our investigation into whether KDM1B, another H3K4me2 demethylase, exerts comparable regulatory effects. Our study identified a strong negative correlation between SOX9 and KDM1B expression in OA cartilage specimens. We identified direct binding of KDM1B to the SOX9 promoter region by chromatin immunoprecipitation and Luciferase reporter assays. Functional studies demonstrated that KDM1B-mediated removal of H3K4me2 modifications significantly suppressed SOX9 transcription, establishing a mechanistic connection between KDM1B activity and chondrocyte dysfunction. In addition to H3K4me2, H3K4me1 is a known substrate of KDM1B, but it was not included in further analysis due to inconsistent detection results of H3K4me1 in our previous experiment. Future investigations should explore the potential roles of H3K4me1 and other histone marks in KDM1B-mediated epigenetic regulation, which may provide additional insights into the complex pathogenesis of OA.

The dynamic regulation of RNA modifications has emerged as a fundamental biological mechanism governing cellular homeostasis and gene expression control [34]. Emerging evidence has highlighted the aberrant RNA modification patterns contribute to disease pathogenesis, including OA [35, 36]. Technological breakthroughs in sequencing platforms have enabled systematic characterization of diverse RNA modifications, such as N6-methyladenosine (m6A), 5-methylcytidine (m5C), and N4-acetylcytidine (ac4C), which collectively modulate critical mRNA metabolism through stability modulation, translational control, and subcellular trafficking [37]. Among these, ac4C has been identified as a highly conserved mRNA modification by NAT10, which has gained attention for its regulatory roles in cellular physiology and disease states. As the sole identified “writer” enzyme for ac4C within the GNAT superfamily, NAT10 combines RNA-binding capacity with acetyltransferase activity to execute post-transcriptional modifications [15]. Accumulating evidence suggests that NAT10-mediated ac4C modification is involved in the pathogenesis of various diseases, such as osteogenesis [38], myocardial infarction [39], tumor progression [40, 41], cardiac remodeling [42], and inflammation [43]. Despite these advances, the functional significance of ac4C modification in OA pathogenesis remained unclear. In this study, our results reveal that NAT10–dependent ac4C deposition enhances KDM1B mRNA stability, thereby contributing to the abnormal upregulation of KDM1B in OA cartilage tissues and chondrocytes. This finding addresses a critical gap in the understanding of RNA modifications in OA progression and elucidates a novel pathogenic mechanism. Importantly, functional experiments have demonstrated that NAT10 suppression alleviates chondrocyte dysfunction by modulation of the KDM1B/SOX9 pathway, highlighting the therapeutic potential of targeting NAT10-mediated ac4C modification for OA treatment.

Notwithstanding these significant findings, our study has certain limitations that warrant attention. First, our multiscale approach confirmed NAT10/KDM1B/SOX9 interactions across cellular and tissue contexts, but the involvement of this axis in NAT10-promoted OA cartilage damage has only been demonstrated in vitro and requires further validation in animal models. Second, although intra-articular treatment with aav-shKDM1B significantly improved synovitis in OA mice, the precise mechanistic cascade—particularly regarding immune cell modulation versus direct chondroprotection—requires elucidation through spatial transcriptomics and cell-type-specific knockout models. Furthermore, the clinical correlation analysis was limited to existing GEO datasets and a single patient cohort, future multicenter studies with longitudinal OA samples are required to validate the stage-dependent dynamics of NAT10/KDM1B/SOX9 expression in human disease progression. Addressing these limitations in our future investigations will provide a more comprehensive understanding of the NAT10/ac4C/KDM1B/SOX9 axis and its therapeutic potential.

## Conclusion

Our study reveals a novel pathogenic mechanism in OA, wherein NAT10-mediated ac4C modification enhances KDM1B mRNA stability through sequence-specific acetylation, driving its pathological accumulation in OA cartilage and chondrocytes. The upregulated KDM1B subsequently binds to the SOX9 promoter and catalyzes H3K4me2 demethylation to suppress SOX9 expression, resulting in chondrocyte proliferation inhibition coupled with ECM metabolic homeostatic disruption, thereby accelerating OA progression (Fig. 8E). These findings not only uncover a novel molecular mechanism driving OA pathogenesis but also highlights the NAT10/ac4C/KDM1B/SOX9 axis as a promising therapeutic target. Notably, this newly epigenetic pathway represents an independent yet complementary avenue to our prior research on the transcription factor-centric ETS2/STAT1/miR-155 axis in OA pathogenesis. Developed concurrently via distinct mechanisms—histone modification and transcriptional regulation—these two pathways collectively broaden the scope for OA therapeutic development. Future studies should validate these findings in vivo, explore the translational applications of targeting the NAT10/ac4C/KDM1B/SOX9 axis, and critically examine potential crosstalk between this epigenetic axis and the ETS2/STAT1/miR-155 transcriptional network to facilitate the development of effective multi-targeted OA therapeutics.

## Supplementary Information

Supplementary Figure 1 The expression levels of SETD4 and PRMT3 in normal and OA cartilage tissues were analyzed based on multiple GEO datasets. **A **The expression levels of SETD4 in 7 normal and 8 OA cartilage samples from GSE168505 and GSE178557 datasets. **B **The expression levels of PRMT3 in 7 normal and 8 OA cartilage samples from GSE168505 and GSE178557 datasets. **C **The expression levels of SETD4 and PRMT3 in 18 normal and 20 OA cartilage samples from GSE114007 datasets. OA, osteoarthritis cartilage; Normal, non-OA cartilage. ^n.s^.*p* > 0.05, ^#^*p* < 0.05, ^##^*p* < 0.01, and ^###^*p* < 0.001 (PNG 305 KB)

Supplementary file1 (TIF 6.08 MB)

Supplementary Figure 2 The infection efficiency of adenovirus was confirmed. **A **RT-qPCR analysis showed that KDM1B expression was significantly increased in chondrocytes infected with adv-KDM1B compared to those infected with adv-NC. **B **RT-qPCR analysis demonstrated that adv-shKDM1B significantly reduced KDM1B mRNA expression in chondrocytes compared to those infected with adv-shNC. adv-NC, negative control adenovirus; adv-KDM1B, KDM1B-overexpressing adenovirus; adv-shNC, negative control short hairpin RNA adenovirus; adv-shKDM1B, KDM1B short hairpin RNA adenovirus. *N* = 3, ^###^*p* < 0.001 (PNG 104 KB)

Supplementary file2 (TIF 2.17 MB)

Supplementary Figure 3 Chondrocytes infected with adv-KDM1B exhibited enhanced IL-1β-induced KDM1B expression, whereas those infected with adv-shKDM1B showed suppressed expression. **A **Western blot analysis assessed the effect of adv-KDM1B or adv-NC transfection on IL-1β-induced KDM1B protein levels in chondrocytes. **B **Western blot was used to analyze the effect of adv-shKDM1B or adv-shNC transfection on IL-1β-induced KDM1B expression in chondrocytes. adv-NC, negative control adenovirus; adv-KDM1B, KDM1B-overexpressing adenovirus; adv-shNC, negative control short hairpin RNA adenovirus; adv-shKDM1B, KDM1B short hairpin RNA adenovirus. *N* = 3, ^n.s^.*p* > 0.05, ^#^*p* < 0.05, and ^###^*p* < 0.0011 (PNG 150 KB)

Supplementary file3 (TIF 3.35 MB)

Supplementary Fig. 4 Role of KDM1B in IL-1β-induced chondrocyte proliferation inhibition. **A **Western blot assessed Ki67, p21, and p27 protein levels in chondrocytes treated with IL-1β or adv-KDM1B. **B **The protein expression levels of Ki67, p21, and p27 in chondrocytes treated with IL-1β or adv-shKDM1B were detected by western blot. adv-NC, negative control adenovirus; adv-KDM1B, KDM1B-overexpressing adenovirus; adv-shNC, negative control short hairpin RNA adenovirus; adv-shKDM1B, KDM1B short hairpin RNA adenovirus. *N* = 3, ^#^*p* < 0.05, ^##^*p* < 0.01, and ^###^*p* < 0.001 (PNG 405 KB)

Supplementary file4 (TIF 8.61 MB)

Supplementary Fig. 5 Knockdown of KDM1B significantly alleviates pathological synovitis in DMM-induced OA mice. **A **Representative HE staining of synovial tissues from the indicated mice. Scale bars = 50μm. **B **Synovium scores was measured in synovial tissue from the indicated mice. **C**Quantitative immunofluorescence analysis of synovial F4/80^+^ macrophage infiltration and iNOS^+^ M1 polarization across experimental mouse cohorts. **D **Representative immunofluorescence micrographs of synovial membrane sections stained for macrophage marker F4/80 (green) and M1 polarization marker iNOS (pink). Nuclei counterstained with DAPI (blue), Scale bar = 50μm. DMM, destabilizing the medial meniscus-induced OA model; aav-shNC, negative control short hairpin RNA adeno-associated virus; aav-shKDM1B, KDM1B short hairpin RNA adeno-associated virus. *N* = 6, ^#^*p* < 0.05, ^##^*p* < 0.01, and ^###^*p* < 0.001 (PNG 3.46 MB)

Supplementary file5 (TIF 18.1 MB)

Supplementary Fig. 6 NAT10 knockdown inhibits IL-1β-induced KDM1B expression and ECM metabolism imbalance in chondrocytes. **A **The protein expression of Flag-NAT10 was measured by western blot in chondrocytes transfected with adv-NAT10-wt or adv-NAT10-mut. **B **RT-qPCR and western blotting were used to assess NAT10 mRNA and protein levels in chondrocytes treated with IL-1β or infected with adv-shNAT10. **C **The protein and mRNA expression levels of KDM1B were detected by RT-qPCR and western blot in chondrocytes treated with IL-1β or infected with adv-shNAT10. **D-E.** The protein expression levels of extracellular matrix metabolic markers (MMP13, ADAMTS5, Collagen II, and Aggrecan) were evaluated by western blot in chondrocytes IL-1β-stimulated chondrocytes post-NAT10 knockdown. adv-shNC, negative control short hairpin RNA adenovirus; adv-shNAT10, NAT10 short hairpin RNA adenovirus. *N* = 3, ^#^*p* < 0.05, ^##^*p* < 0.01, and ^###^*p* < 0.001 (PNG 692 KB)

Supplementary file6 (TIF 18.5 MB)

Supplementary Fig. 7 The relationship between KDM1B and SOX9. **A **Analysis of the GSE114007 dataset revealed significant downregulation of SOX9 expression in human OA cartilage tissues (*n* = 20) compared to normal controls (*n* = 18). And Spearman correlation analysis further demonstrated a significant inverse correlation between SOX9 and KDM1B expression levels. **B **RT-qPCR analysis of human OA cartilage specimens revealed significantly downregulated SOX9 mRNA levels in grade III/IV tissues (*n* = 25) compared to grade I/II tissues (*n* = 10). And Spearman correlation analysis further demonstrated a significant inverse correlation between SOX9 and KDM1B expression levels. **C **The mRNA expression levels of SOX9 in cartilage tissue of OA mice treated with aav-shKDM1B were evaluated by RT-qPCR. **D **The protein expression levels of SOX9 and H3K4me2 in the indicated cartilage tissue were evaluated by western blot. Normal, non-OA cartilage; OA, osteoarthritis cartilage; DMM, destabilizing the medial meniscus-induced OA mice; aav-shNC, negative control short hairpin RNA adeno-associated virus; aav-shKDM1B, KDM1B short hairpin RNA adeno-associated virus. *N* = 3 ~ 6, ^#^*p* < 0.05, ^##^*p* < 0.01, and ^###^*p* < 0.001 (PNG 682 KB)

Supplementary file7 (TIF 15.6 MB)

Supplementary Fig. 8 KDM1B knockdown rescues experimental synovitis in DMM-induced OA mice through SOX9 upregulation. **A **Representative images of H&E staining in the indicated synovial tissues. Scale bars = 50μm. **B **Synovium scores was measured in synovial tissue from the indicated mice. **C **Quantitative immunofluorescence analysis of synovial F4/80^+^ macrophage infiltration and iNOS^+^ M1 polarization across experimental mouse cohorts. **D **Representative immunofluorescence micrographs of synovial membrane sections stained for macrophage marker F4/80 (green) and M1 polarization marker iNOS (pink). Nuclei counterstained with DAPI (blue), Scale bar = 50μm. DMM, destabilizing the medial meniscus-induced OA mice; aav-shNC, negative control short hairpin RNA adeno-associated virus; aav-shKDM1B, KDM1B short hairpin RNA adeno-associated virus; aav-shSOX9, SOX9 short hairpin RNA adeno-associated virus. *N* = 6, ^#^*p* < 0.05, ^##^*p* < 0.01, and ^###^*p* < 0.001 (PNG 5.00 MB)

Supplementary file8 (TIF 19.8 MB)

Supplementary file4 (DOCX 30.4 KB)

## Data Availability

The datasets used and/or analyzed during the current study are available from the corresponding author on reasonable request.

## References

[1] Quicke JG, Conaghan PG, Corp N, Peat G (2022) Osteoarthritis year in review 2021: epidemiology & therapy. Osteoarthr Cartil 30:196–206 10.1016/j.joca.2021.10.003 34695571

[2] Emami A, Namdari H, Parvizpour F, Arabpour Z (2023) Challenges in osteoarthritis treatment. Tissue Cell 80:101992 36462384 10.1016/j.tice.2022.101992

[3] Zhou ZB, Huang GX, Fu Q, Han B, Lu JJ, Chen AM, Zhu L (2019) Circrna.33186 contributes to the pathogenesis of osteoarthritis by sponging miR-127-5p. Mol Therapy: J Am Soc Gene Therapy 27:531–541 10.1016/j.ymthe.2019.01.006 PMC6402950 30692016

[4] Shen P, Gao J, Huang S, You C, Wang H, Chen P, Yao T, Gao T, Zhou B, Shen S, Zhao X, Ma J (2023) LncRNA AC006064.4-201 serves as a novel molecular marker in alleviating cartilage senescence and protecting against osteoarthritis by destabilizing CDKN1B mRNA via interacting with PTBP1. Biomark Res 11:39 37055817 10.1186/s40364-023-00477-6 PMC10099822

[5] Huang H, Yan J, Lan X, Guo Y, Sun M, Zhao Y, Zhang F, Sun J, Lu S (2023) LncRNA WDR11-AS1 promotes extracellular matrix synthesis in osteoarthritis by directly interacting with RNA-binding protein PABPC1 to stabilize SOX9 expression. Int J Mol Sci. 24(1):817 10.3390/ijms24010817 36614257 10.3390/ijms24010817 PMC9820994

[6] Núñez-Carro C, Blanco-Blanco M, Villagrán-Andrade KM, Blanco FJ, de Andrés MC (2023) Epigenetics as a therapeutic target in osteoarthritis. Pharmaceuticals. 16(2):156. 10.3390/ph16020156 37259307 10.3390/ph16020156 PMC9964205

[7] Tong L, Yu H, Huang X, Shen J, Xiao G, Chen L, Wang H, Xing L, Chen D (2022) Current understanding of osteoarthritis pathogenesis and relevant new approaches. Bone Res 10:60 36127328 10.1038/s41413-022-00226-9 PMC9489702

[8] Cai Z, Long T, Zhao Y, Lin R, Wang Y (2022) Epigenetic regulation in knee osteoarthritis. Front Genet 13:942982 35873487 10.3389/fgene.2022.942982 PMC9304589

[9] Hou C, Ye Z, Yang S, Jiang Z, Wang J, Wang E (2022) Lysine demethylase 1B (Kdm1b) enhances somatic reprogramming through inducing pluripotent gene expression and promoting cell proliferation. Exp Cell Res 420:113339 36075448 10.1016/j.yexcr.2022.113339

[10] Musella M, Guarracino A, Manduca N, Galassi C, Ruggiero E, Potenza A, Maccafeo E, Manic G, Mattiello L, Soliman Abdel Rehim S, Signore M, Pietrosanto M, Helmer-Citterich M, Pallocca M, Fanciulli M, Bruno T, De Nicola F, Corleone G, Di Benedetto A, Ercolani C, Pescarmona E, Pizzuti L, Guidi F, Sperati F, Vitale S, Macchia D, Spada M, Schiavoni G, Mattei F, De Ninno A, Businaro L, Lucarini V, Bracci L, Aricò E, Ziccheddu G, Facchiano F, Rossi S, Sanchez M, Boe A, Biffoni M, De Maria R, Vitale I, Sistigu A (2022) Type I IFNs promote cancer cell stemness by triggering the epigenetic regulator KDM1B. Nat Immunol 23:1379–1392 36002648 10.1038/s41590-022-01290-3 PMC9477743

[11] Anvar Z, Chakchouk I, Sharif M, Mahadevan S, Nasiotis ET, Su L, Liu Z, Wan YW, Van den Veyver IB (2023) Loss of the maternal effect gene Nlrp2 alters the transcriptome of ovulated mouse oocytes and impacts expression of histone demethylase KDM1B. Reprod Sci 30:2780–2793 36976514 10.1007/s43032-023-01218-8 PMC10524210

[12] Liu S, Tang S, Yang G, Li Q (2023) Lysine demethylase 1B promotes tear secretion disorder in sjogren’s syndrome by regulating the PAX6/CLU axis. J Mol Neuroscience: MN 73:28–38 10.1007/s12031-022-02094-8 36542318

[13] Chen X, Hao Y, Liu Y, Zhong S, You Y, Ao K, Chong T, Luo X, Yin M, Ye M, He H, Lu A, Chen J, Li X, Zhang J, Guo X (2023) NAT10/ac4C/FOXP1 promotes malignant progression and facilitates immunosuppression by reprogramming glycolytic metabolism in cervical cancer. Adv Sci 10:e2302705 10.1002/advs.202302705 PMC10646273 37818745

[14] Arango D, Sturgill D, Alhusaini N, Dillman AA, Sweet TJ, Hanson G, Hosogane M, Sinclair WR, Nanan KK, Mandler MD, Fox SD, Zengeya TT, Andresson T, Meier JL, Coller J, Oberdoerffer S (2018) Acetylation of cytidine in mRNA promotes translation efficiency. Cell 175:1872–1886e24 30449621 10.1016/j.cell.2018.10.030 PMC6295233

[15] Xie L, Zhong X, Cao W, Liu J, Zu X, Chen L (2023) Mechanisms of NAT10 as ac4c writer in diseases. Mol Ther Nucleic Acids 32:359–368 37128278 10.1016/j.omtn.2023.03.023 PMC10148080

[16] Zhang Y, Lei Y, Dong Y, Chen S, Sun S, Zhou F, Zhao Z, Chen B, Wei L, Chen J, Meng Z (2024) Emerging roles of RNA ac4c modification and NAT10 in mammalian development and human diseases. Pharmacol Ther 253:108576 38065232 10.1016/j.pharmthera.2023.108576

[17] Kloppenburg M, Kroon FP, Blanco FJ, Doherty M, Dziedzic KS, Greibrokk E, Haugen IK, Herrero-Beaumont G, Jonsson H, Kjeken I, Maheu E, Ramonda R, Ritt MJ, Smeets W, Smolen JS, Stamm TA, Szekanecz Z, Wittoek R, Carmona L (2019) 2018 update of the EULAR recommendations for the management of hand osteoarthritis. Ann Rheum Dis 78:16–24 30154087 10.1136/annrheumdis-2018-213826

[18] Chen S, Zhu X, Ou W, Kang L, Situ J, Liao Z, Huang L, Qi W, Ni S (2023) ETS2 overexpression ameliorates cartilage injury in osteoarthritis by the ETS2/miR-155/STAT1/DNMT1 feedback loop pathway. Biochimica et Biophysica Acta (BBA) 1866:194965 10.1016/j.bbagrm.2023.194965 37524226

[19] Lin J, Xiang Y, Huang J, Zeng H, Zeng Y, Liu J, Wu T, Liang Q, Liang X, Li J, Zhou C (2022) NAT10 maintains OGA mRNA stability through ac4C modification in regulating oocyte maturation. Front Endocrinol 13:907286 10.3389/fendo.2022.907286 PMC9352860 35937804

[20] Yu XM, Li SJ, Yao ZT, Xu JJ, Zheng CC, Liu ZC, Ding PB, Jiang ZL, Wei X, Zhao LP, Shi XY, Li ZG, Xu WW, Li B (2023) N4-acetylcytidine modification of LncRNA CTC-490G23.2 promotes cancer metastasis through interacting with PTBP1 to increase CD44 alternative splicing. Oncogene 42:1101–1116 36792757 10.1038/s41388-023-02628-3

[21] Wu Y, Shen S, Chen J, Ni W, Wang Q, Zhou H, Chen J, Zhang H, Mei Z, Sun X, Shen P, Jie Z, Xu W, Hong Z, Ma Y, Wang K, Wan S, Wu H, Xie Z, Qin A, Fan S (2023) Metabolite asymmetric dimethylarginine (ADMA) functions as a destabilization enhancer of SOX9 mediated by DDAH1 in osteoarthritis. Sci Adv 9:eade5584 36753544 10.1126/sciadv.ade5584 PMC9908022

[22] Carmon I, Zecharyahu L, Elayyan J, Meka SRK, Reich E, Kandel L, Bilkei-Gorzo A, Zimmer A, Mechoulam R, Kravchenko-Balasha N, Dvir-Ginzberg M (2023) HU308 mitigates osteoarthritis by stimulating Sox9-related networks of carbohydrate metabolism. J Bone Mineral Research: Official J Am Soc Bone Mineral Res 38:154–170 10.1002/jbmr.4741 PMC10098743 36350089

[23] Song H, Park KH (2020) Regulation and function of SOX9 during cartilage development and regeneration. Semin Cancer Biol 67:12–23 32380234 10.1016/j.semcancer.2020.04.008

[24] Zhang M, Lu Q, Miller AH, Barnthouse NC, Wang J (2016) Dynamic epigenetic mechanisms regulate age-dependent SOX9 expression in mouse articular cartilage. Int J Biochem Cell Biol 72:125–134 26806292 10.1016/j.biocel.2016.01.013 PMC4762732

[25] Yao Q, Wu X, Tao C, Gong W, Chen M, Qu M, Zhong Y, He T, Chen S (2023) Osteoarthritis: pathogenic signaling pathways and therapeutic targets. Signal Transduct Target Ther 8:56 36737426 10.1038/s41392-023-01330-w PMC9898571

[26] Charidemou E, Koufaris C, Louca M, Kirmizis A, Rubio-Tomás T (2023) Histone methylation in pre-cancerous liver diseases and hepatocellular carcinoma: recent overview. Clin Translational Oncology: Official Publication Federation Span Oncol Soc Natl Cancer Inst Mexico 25:1594–1605 10.1007/s12094-023-03078-9 PMC10203016 36650321

[27] Mao F, Shi YG (2023) Targeting the LSD1/KDM1 family of lysine demethylases in cancer and other human diseases. Adv Exp Med Biol 1433:15–49 37751134 10.1007/978-3-031-38176-8_2

[28] Maes T, Mascaró C, Ortega A, Lunardi S, Ciceri F, Somervaille TC, Buesa C (2015) KDM1 histone lysine demethylases as targets for treatments of oncological and neurodegenerative disease. Epigenomics 7:609–626 26111032 10.2217/epi.15.9

[29] El Mansouri FE, Nebbaki SS, Kapoor M, Afif H, Martel-Pelletier J, Pelletier JP, Benderdour M, Fahmi H (2014) Lysine-specific demethylase 1-mediated demethylation of histone H3 lysine 9 contributes to Interleukin 1β-induced microsomal prostaglandin E synthase 1 expression in human osteoarthritic chondrocytes. Arthritis Res Ther 16:R113 24886859 10.1186/ar4564 PMC4060543

[30] Durand AL, Dufour A, Aubert-Foucher E, Oger-Desfeux C, Pasdeloup M, Lustig S, Servien E, Vaz G, Perrier-Groult E, Mallein-Gerin F, Lafont JE (2020) The lysine specific demethylase-1 negatively regulates the COL9A1 gene in human articular chondrocytes. Int J Mol Sci. 21(17):6322 10.3390/ijms21176322 32878268 10.3390/ijms21176322 PMC7504057

[31] Lefebvre V, Angelozzi M, Haseeb A (2019) SOX9 in cartilage development and disease. Curr Opin Cell Biol 61:39–47 31382142 10.1016/j.ceb.2019.07.008 PMC6956855

[32] Mao G, Xu Y, Long D, Sun H, Li H, Xin R, Zhang Z, Li Z, Yang Z, Kang Y (2021) Exosome-transported circRNA_0001236 enhances chondrogenesis and suppress cartilage degradation via the miR-3677-3p/Sox9 axis. Stem Cell Res Ther 12:389 34256841 10.1186/s13287-021-02431-5 PMC8278601

[33] Ouyang Y, Wang W, Tu B, Zhu Y, Fan C, Li Y (2019) Overexpression of SOX9 alleviates the progression of human osteoarthritis in vitro and in vivo. Drug Des Devel Ther 13:2833–2842 31496660 10.2147/DDDT.S203974 PMC6698167

[34] Wang C, Hou X, Guan Q, Zhou H, Zhou L, Liu L, Liu J, Li F, Li W, Liu H (2023) RNA modification in cardiovascular disease: implications for therapeutic interventions. Signal Transduct Target Ther 8:412 37884527 10.1038/s41392-023-01638-7 PMC10603151

[35] Yu Y, Lu S, Liu X, Li Y, Xu J (2023) Identification and analysis of RNA-5-methylcytosine-related key genes in osteoarthritis. BMC Genomics 24:539 37700248 10.1186/s12864-023-09651-4 PMC10496305

[36] Lin Z, Jiang T, Zheng W, Zhang J, Li A, Lu C, Liu W (2023) N6-methyladenosine (m6A) methyltransferase WTAP-mediated miR-92b-5p accelerates osteoarthritis progression. Cell Commun Signal 21(1):199 37563688 10.1186/s12964-023-01228-8 PMC10416510

[37] Jin C, Wang T, Zhang D, Yang P, Zhang C, Peng W, Jin K, Wang L, Zhou J, Peng C, Tan Y, Ji J, Chen Z, Sun Q, Yang S, Tang J, Feng Y, Sun Y (2022) Acetyltransferase NAT10 regulates the Wnt/β-catenin signaling pathway to promote colorectal cancer progression via ac(4)C acetylation of KIF23 mRNA. J Experimental Clin Cancer Research: CR 41:345 10.1186/s13046-022-02551-7 PMC9753290 36522719

[38] Yang W, Li HY, Wu YF, Mi RJ, Liu WZ, Shen X, Lu YX, Jiang YH, Ma MJ, Shen HY (2021) Ac4C acetylation of RUNX2 catalyzed by NAT10 spurs osteogenesis of BMSCs and prevents ovariectomy-induced bone loss. Mol Ther Nucleic Acids 26:135–147 34513300 10.1016/j.omtn.2021.06.022 PMC8413676

[39] Wang K, Zhou LY, Liu F, Lin L, Ju J, Tian PC, Liu CY, Li XM, Chen XZ, Wang T, Wang F, Wang SC, Zhang J, Zhang YH, Tian JW, Wang K (2022) Piwi-interacting RNA HAAPIR regulates cardiomyocyte death after myocardial infarction by promoting NAT10-mediated ac(4) c acetylation of Tfec mRNA. Advanced science (Weinheim, Baden-Wurttemberg, Germany) 9:e2106058 35138696 10.1002/advs.202106058 PMC8922123

[40] Liao L, He Y, Li SJ, Yu XM, Liu ZC, Liang YY, Yang H, Yang J, Zhang GG, Deng CM, Wei X, Zhu YD, Xu TY, Zheng CC, Cheng C, Li A, Li ZG, Liu JB, Li B (2023) Lysine 2-hydroxyisobutyrylation of NAT10 promotes cancer metastasis in an ac4C-dependent manner. Cell Res 33:355–371 36882514 10.1038/s41422-023-00793-4 PMC10156899

[41] Luo J, Cao J, Chen C, Xie H (2023) Emerging role of RNA acetylation modification ac4C in diseases: current advances and future challenges. Biochem Pharmacol 213:115628 37247745 10.1016/j.bcp.2023.115628

[42] Shi J, Yang C, Zhang J, Zhao K, Li P, Kong C, Wu X, Sun H, Zheng R, Sun W, Chen L, Kong X (2023) NAT10 is involved in cardiac remodeling through ac4C-mediated transcriptomic regulation. Circ Res 133:989–1002 37955115 10.1161/CIRCRESAHA.122.322244

[43] Yan Q, Zhou J, Wang Z, Ding X, Ma X, Li W, Jia X, Gao SJ, Lu C (2023) NAT10-dependent N(4)-acetylcytidine modification mediates PAN RNA stability, KSHV reactivation, and IFI16-related inflammasome activation. Nat Commun 14:6327 37816771 10.1038/s41467-023-42135-3 PMC10564894

