# Supplementary figures and images for: NAT10-mediated ac4C modification of KDM1B drives osteoarthritis progression through epigenetic suppression of SOX9
Source: Cell Mol Life Sci. 2025 Nov 26;82(1):422. doi: 10.1007/s00018-025-05918-z (PMC12647510; doi:10.1007/s00018-025-05918-z)

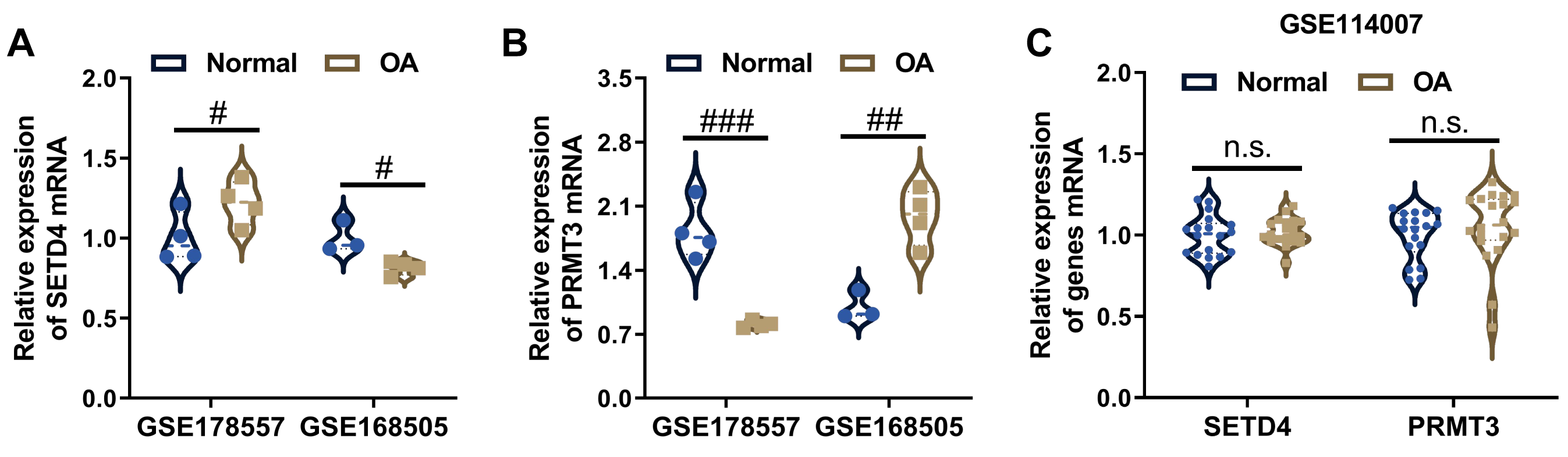

Supplement: Supplementary file 1 — The expression levels of SETD4 and PRMT3 in normal and OA cartilage tissues were analyzed based on multiple GEO datasets. A The expression levels of SETD4 in 7 normal and 8 OA cartilage samples from GSE168505 and GSE178557 datasets. B The expression levels of PRMT3 in 7 normal and 8 OA cartilage samples from GSE168505 and GSE178557 datasets. C The expression levels of SETD4 and PRMT3 in 18 normal and 20 OA cartilage samples from GSE114007 datasets. OA, osteoarthritis cartilage; Normal, non-OA cartilage. n.s.p > 0.05, #p < 0.05, ##p < 0.01, and ###p < 0.001 (PNG 305 KB) [file 18_2025_5918_Fig9_ESM.png]

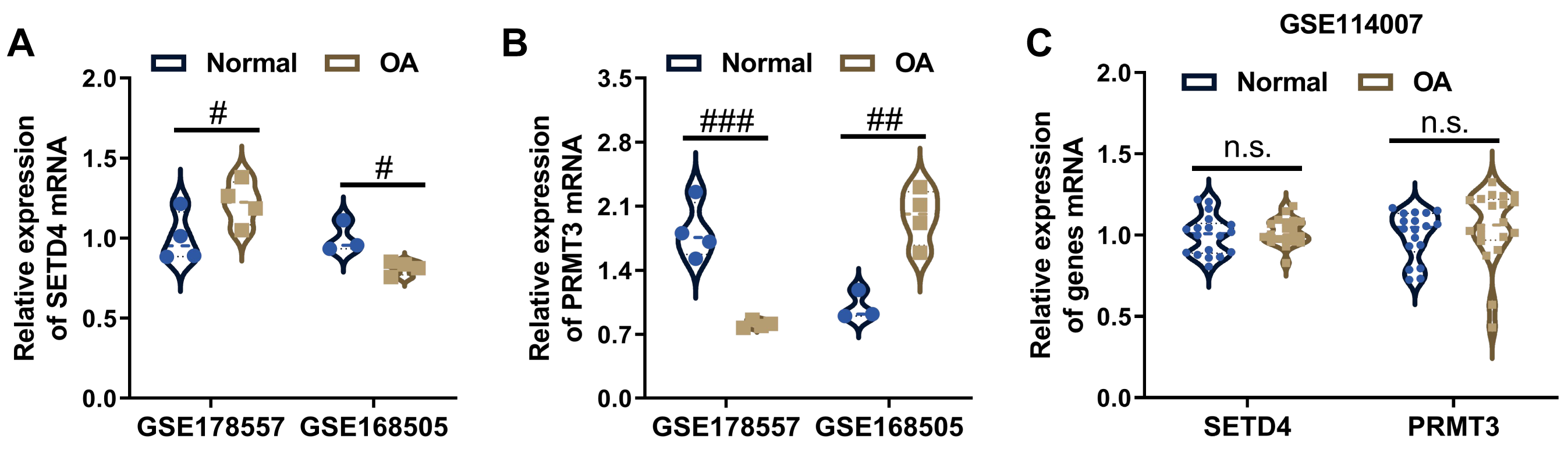

Supplement: Supplementary file 2 — Supplementary file1 (TIF 6.08 MB) [file 18_2025_5918_MOESM1_ESM.tif]

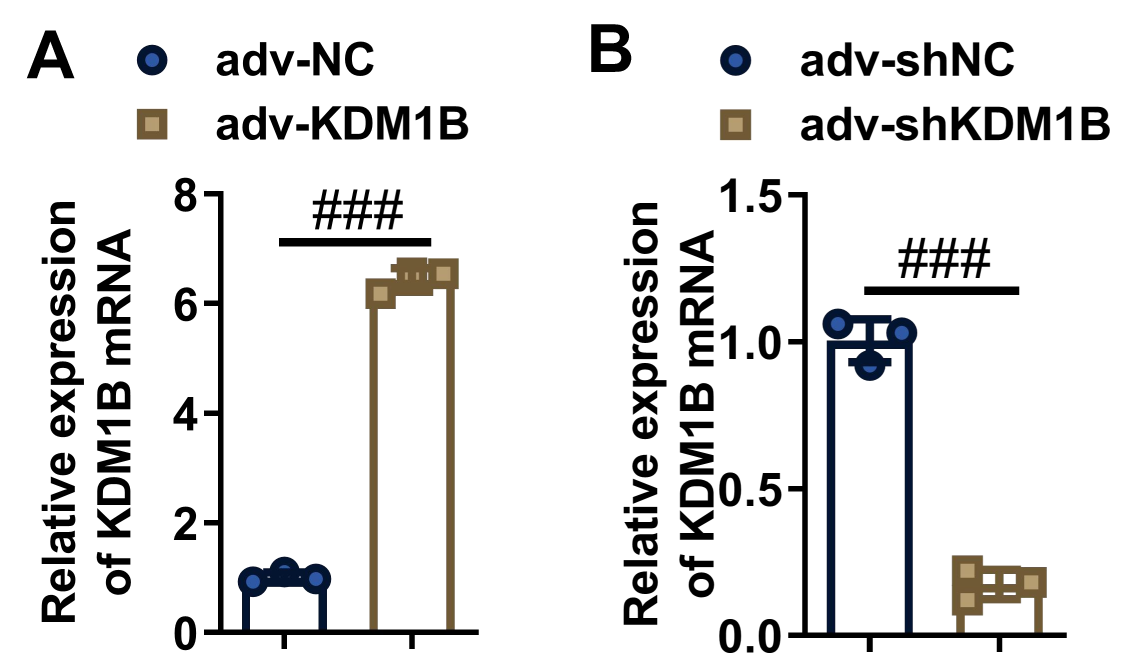

Supplement: Supplementary file 3 — The infection efficiency of adenovirus was confirmed. A RT-qPCR analysis showed that KDM1B expression was significantly increased in chondrocytes infected with adv-KDM1B compared to those infected with adv-NC. B RT-qPCR analysis demonstrated that adv-shKDM1B significantly reduced KDM1B mRNA expression in chondrocytes compared to those infected with adv-shNC. adv-NC, negative control adenovirus; adv-KDM1B, KDM1B-overexpressing adenovirus; adv-shNC, negative control short hairpin RNA adenovirus; adv-shKDM1B, KDM1B short hairpin RNA adenovirus. N = 3, ###p < 0.001 (PNG 104 KB) [file 18_2025_5918_Fig10_ESM.png]

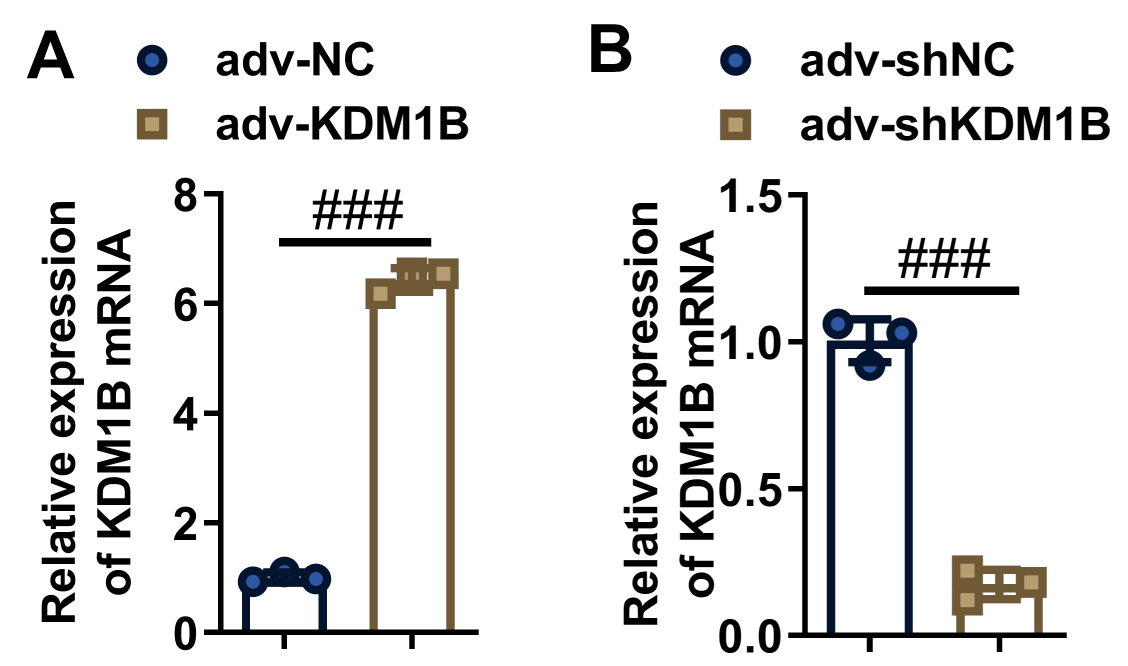

Supplement: Supplementary file 4 — Supplementary file2 (TIF 2.17 MB) [file 18_2025_5918_MOESM2_ESM.tif]

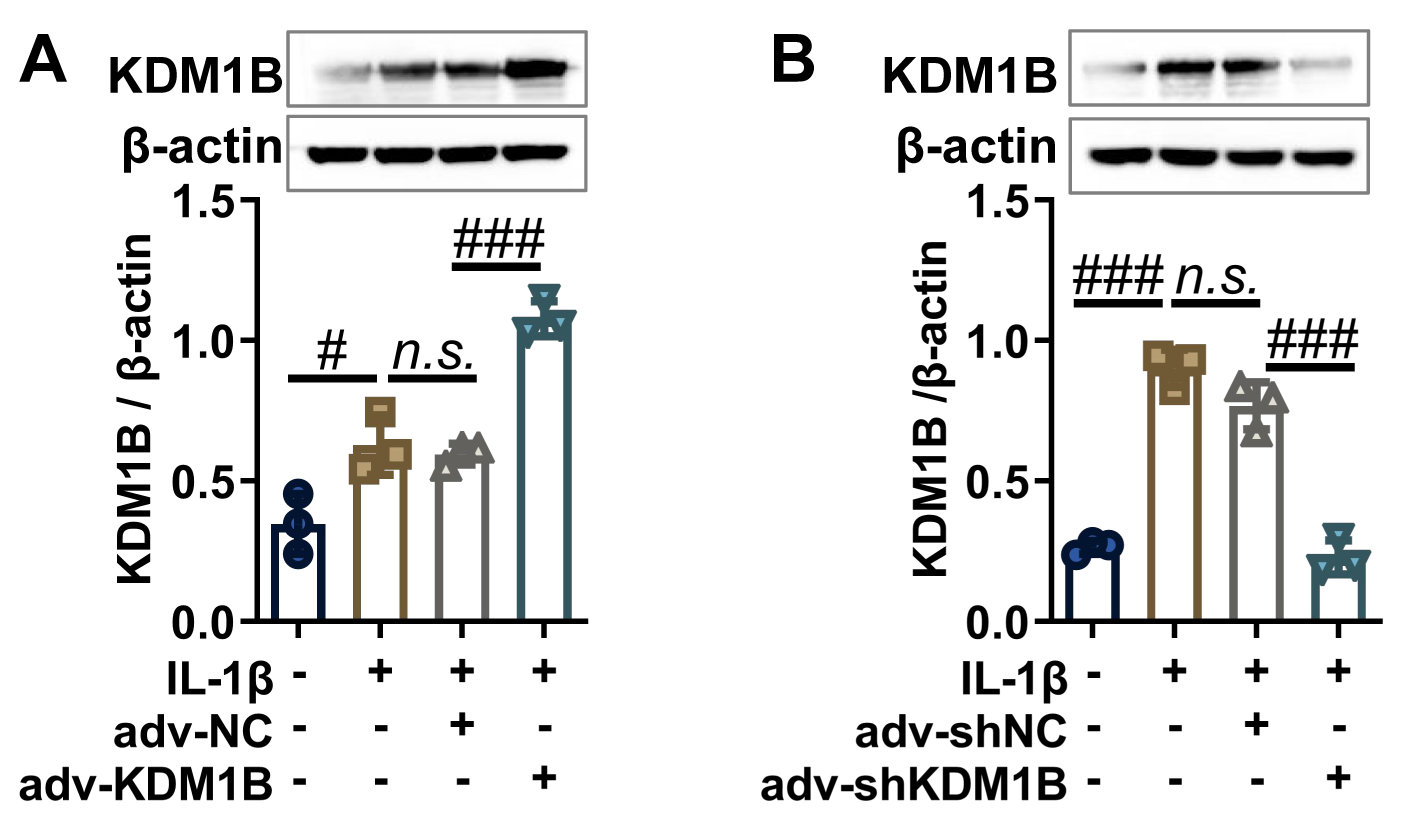

Supplement: Supplementary file 5 — Chondrocytes infected with adv-KDM1B exhibited enhanced IL-1β-induced KDM1B expression, whereas those infected with adv-shKDM1B showed suppressed expression. A Western blot analysis assessed the effect of adv-KDM1B or adv-NC transfection on IL-1β-induced KDM1B protein levels in chondrocytes. B Western blot was used to analyze the effect of adv-shKDM1B or adv-shNC transfection on IL-1β-induced KDM1B expression in chondrocytes. adv-NC, negative control adenovirus; adv-KDM1B, KDM1B-overexpressing adenovirus; adv-shNC, negative control short hairpin RNA adenovirus; adv-shKDM1B, KDM1B short hairpin RNA adenovirus. N = 3, n.s.p > 0.05, #p < 0.05, and ###p < 0.0011 (PNG 150 KB) [file 18_2025_5918_Fig11_ESM.png]

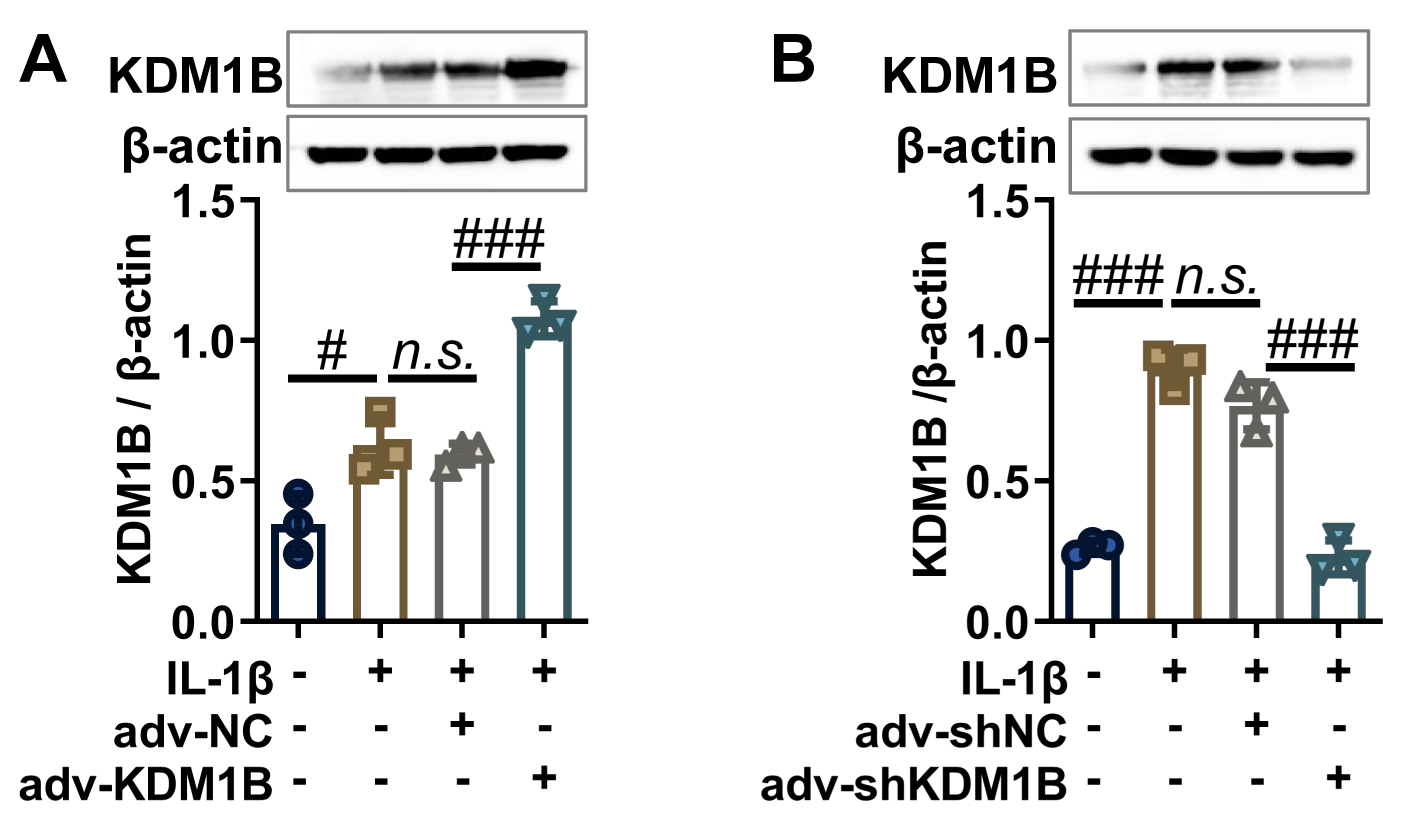

Supplement: Supplementary file 6 — Supplementary file3 (TIF 3.35 MB) [file 18_2025_5918_MOESM3_ESM.tif]

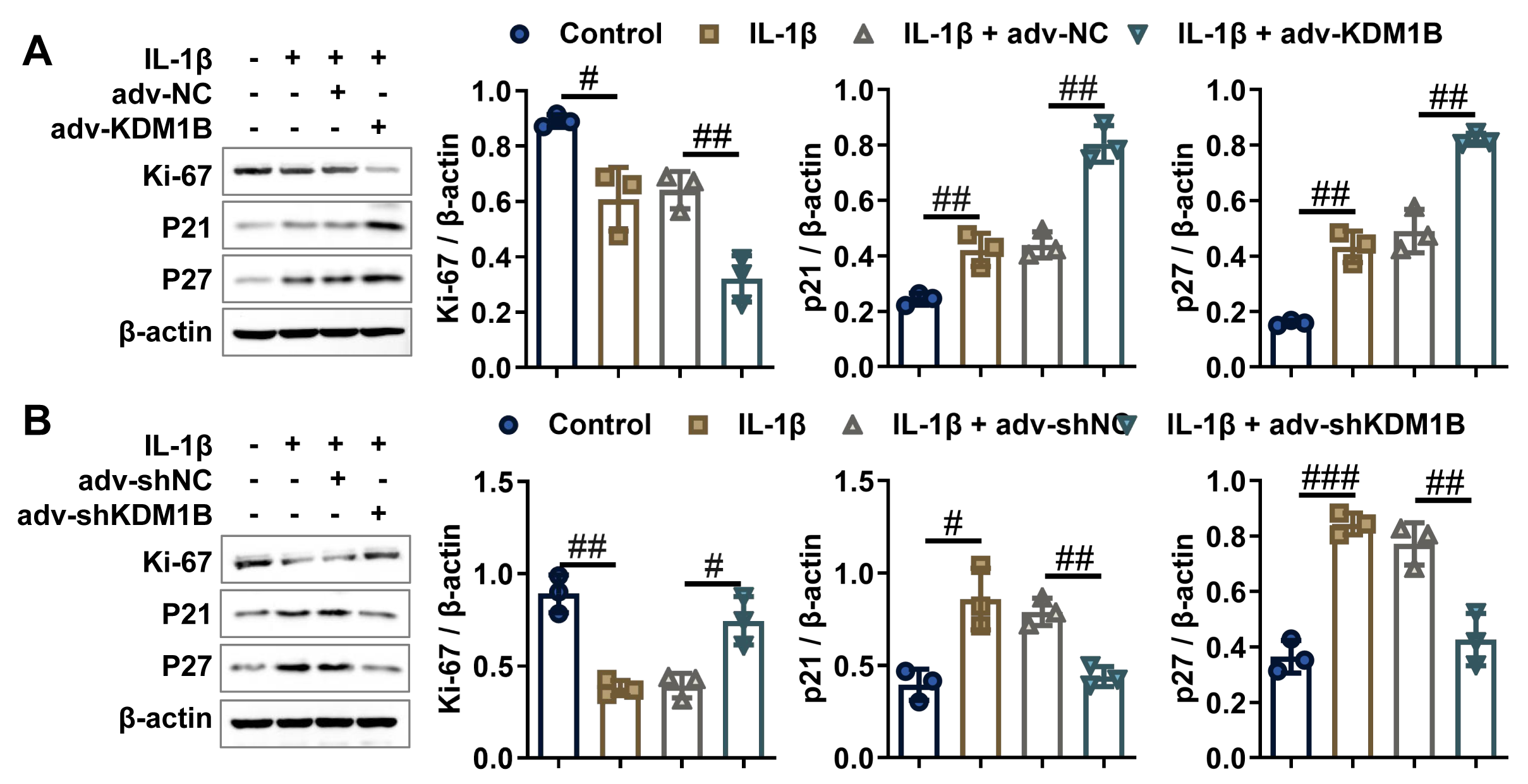

Supplement: Supplementary file 7 — Role of KDM1B in IL-1β-induced chondrocyte proliferation inhibition. A Western blot assessed Ki67, p21, and p27 protein levels in chondrocytes treated with IL-1β or adv-KDM1B. B The protein expression levels of Ki67, p21, and p27 in chondrocytes treated with IL-1β or adv-shKDM1B were detected by western blot. adv-NC, negative control adenovirus; adv-KDM1B, KDM1B-overexpressing adenovirus; adv-shNC, negative control short hairpin RNA adenovirus; adv-shKDM1B, KDM1B short hairpin RNA adenovirus. N = 3, #p < 0.05, ##p < 0.01, and ###p < 0.001 (PNG 405 KB) [file 18_2025_5918_Fig12_ESM.png]

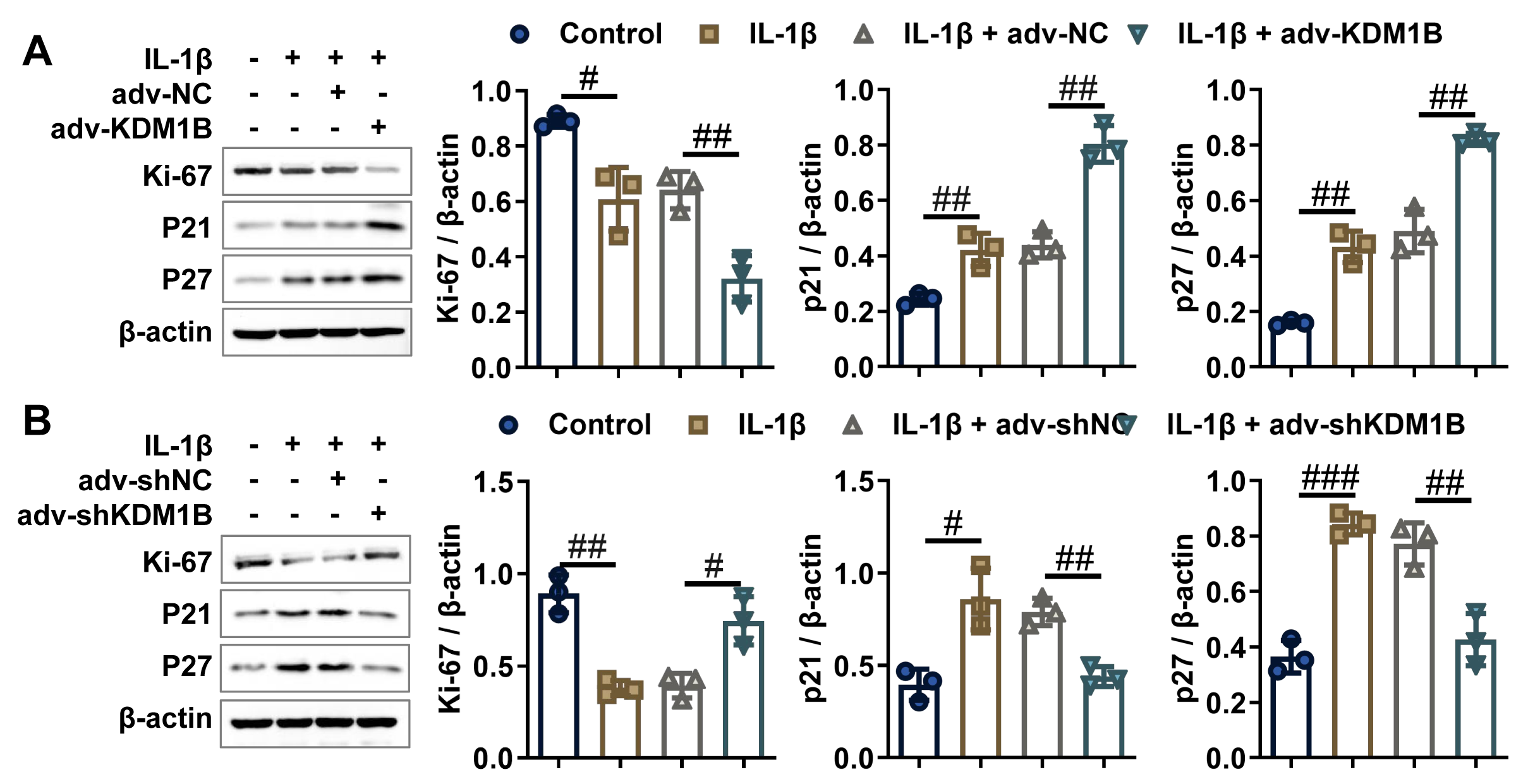

Supplement: Supplementary file 8 — Supplementary file4 (TIF 8.61 MB) [file 18_2025_5918_MOESM4_ESM.tif]

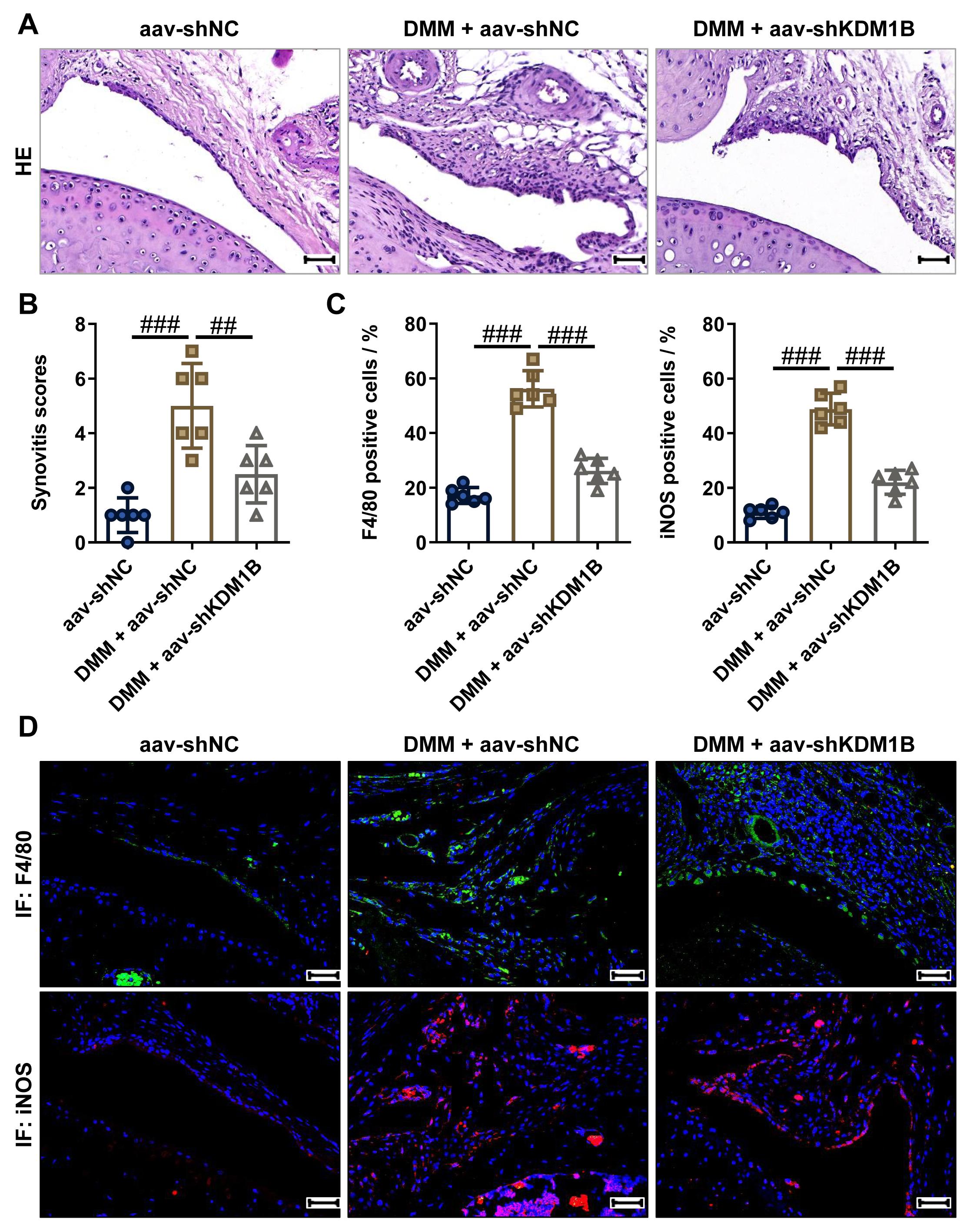

Supplement: Supplementary file 9 — Knockdown of KDM1B significantly alleviates pathological synovitis in DMM-induced OA mice. A Representative HE staining of synovial tissues from the indicated mice. Scale bars = 50μm. B Synovium scores was measured in synovial tissue from the indicated mice. CQuantitative immunofluorescence analysis of synovial F4/80+ macrophage infiltration and iNOS+ M1 polarization across experimental mouse cohorts. D Representative immunofluorescence micrographs of synovial membrane sections stained for macrophage marker F4/80 (green) and M1 polarization marker iNOS (pink). Nuclei counterstained with DAPI (blue), Scale bar = 50μm. DMM, destabilizing the medial meniscus-induced OA model; aav-shNC, negative control short hairpin RNA adeno-associated virus; aav-shKDM1B, KDM1B short hairpin RNA adeno-associated virus. N = 6, #p < 0.05, ##p < 0.01, and ###p < 0.001 (PNG 3.46 MB) [file 18_2025_5918_Fig13_ESM.png]

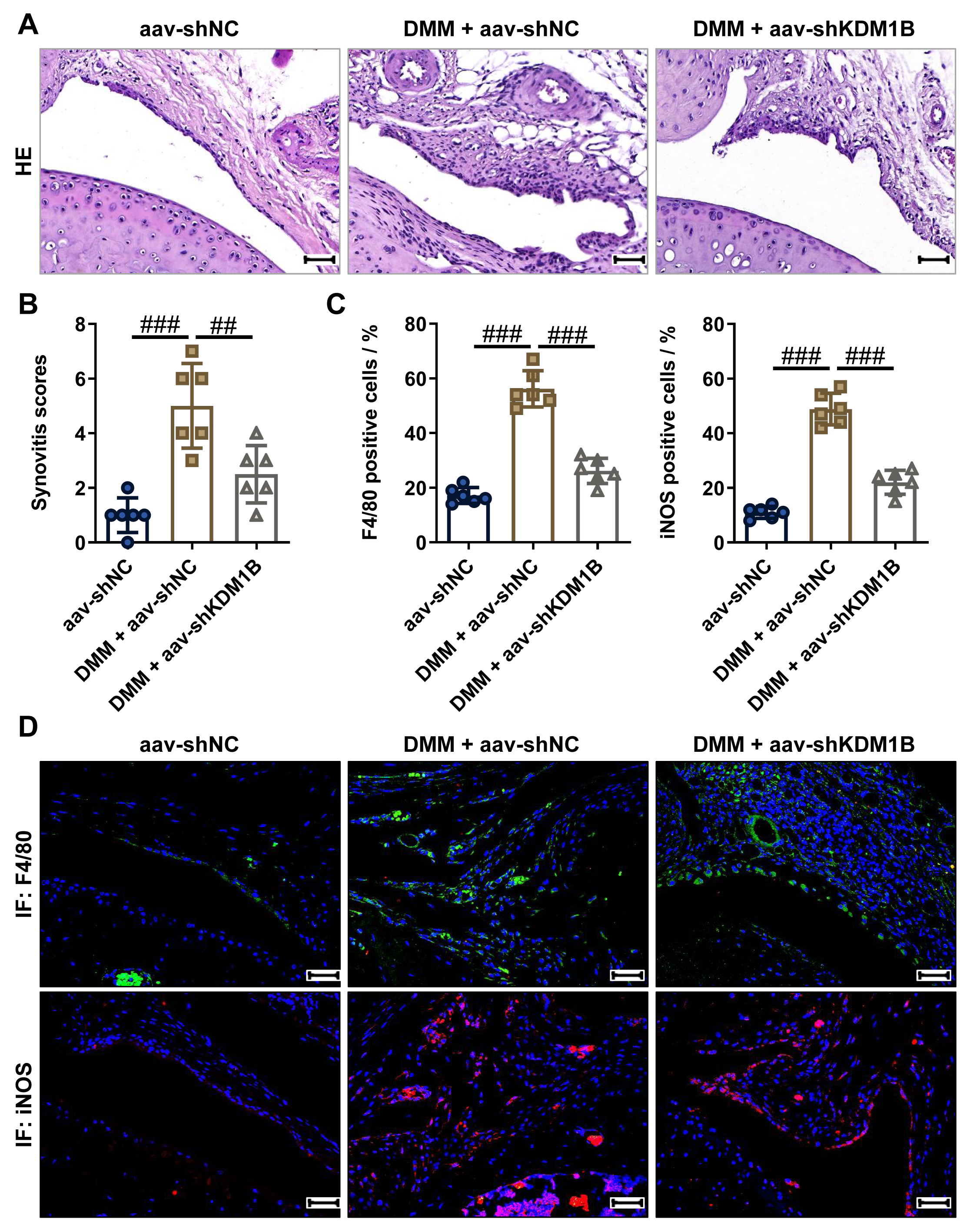

Supplement: Supplementary file 10 — Supplementary file5 (TIF 18.1 MB) [file 18_2025_5918_MOESM5_ESM.tif]

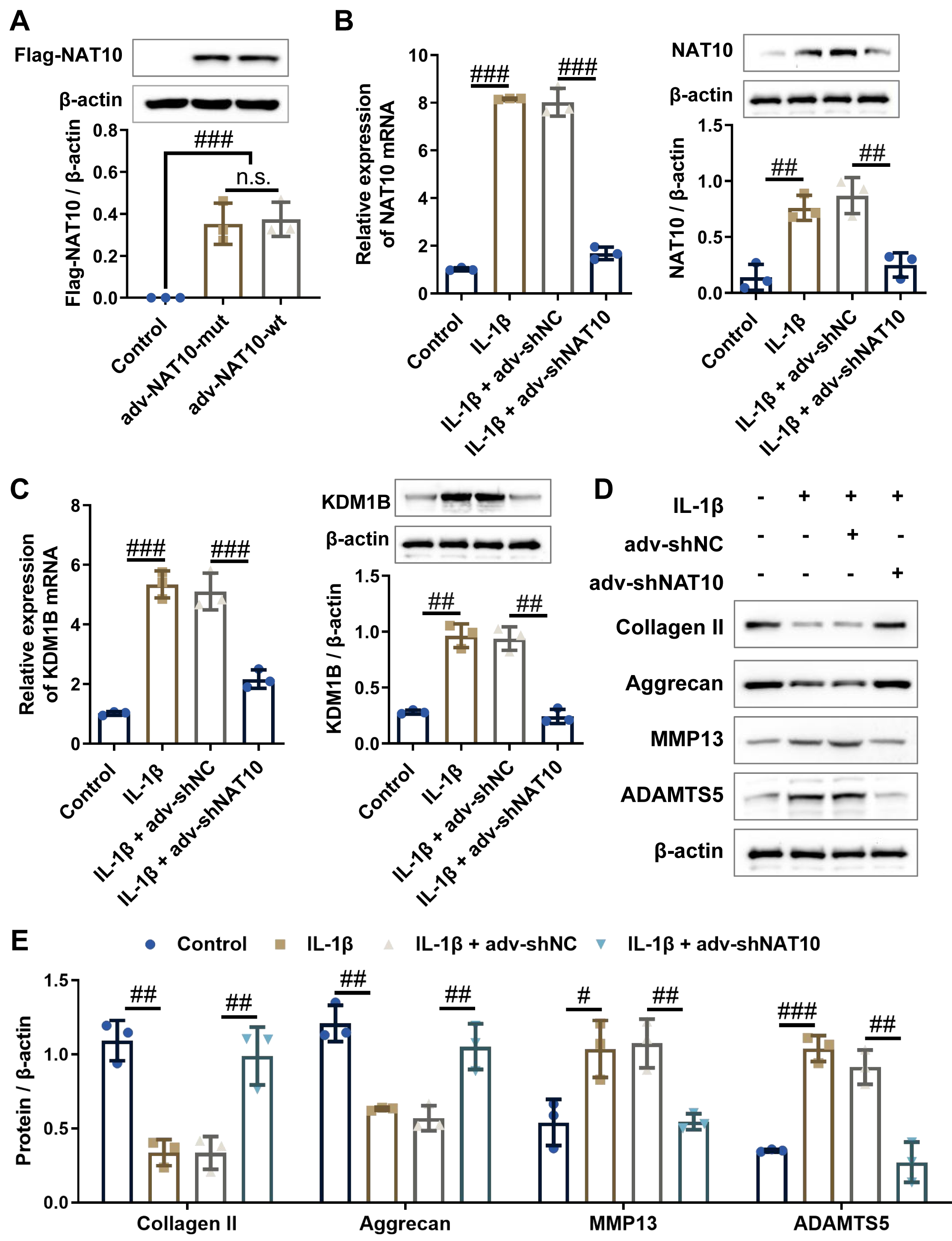

Supplement: Supplementary file 11 — NAT10 knockdown inhibits IL-1β-induced KDM1B expression and ECM metabolism imbalance in chondrocytes. A The protein expression of Flag-NAT10 was measured by western blot in chondrocytes transfected with adv-NAT10-wt or adv-NAT10-mut. B RT-qPCR and western blotting were used to assess NAT10 mRNA and protein levels in chondrocytes treated with IL-1β or infected with adv-shNAT10. C The protein and mRNA expression levels of KDM1B were detected by RT-qPCR and western blot in chondrocytes treated with IL-1β or infected with adv-shNAT10. D-E. The protein expression levels of extracellular matrix metabolic markers (MMP13, ADAMTS5, Collagen II, and Aggrecan) were evaluated by western blot in chondrocytes IL-1β-stimulated chondrocytes post-NAT10 knockdown. adv-shNC, negative control short hairpin RNA adenovirus; adv-shNAT10, NAT10 short hairpin RNA adenovirus. N = 3, #p < 0.05, ##p < 0.01, and ###p < 0.001 (PNG 692 KB) [file 18_2025_5918_Fig14_ESM.png]

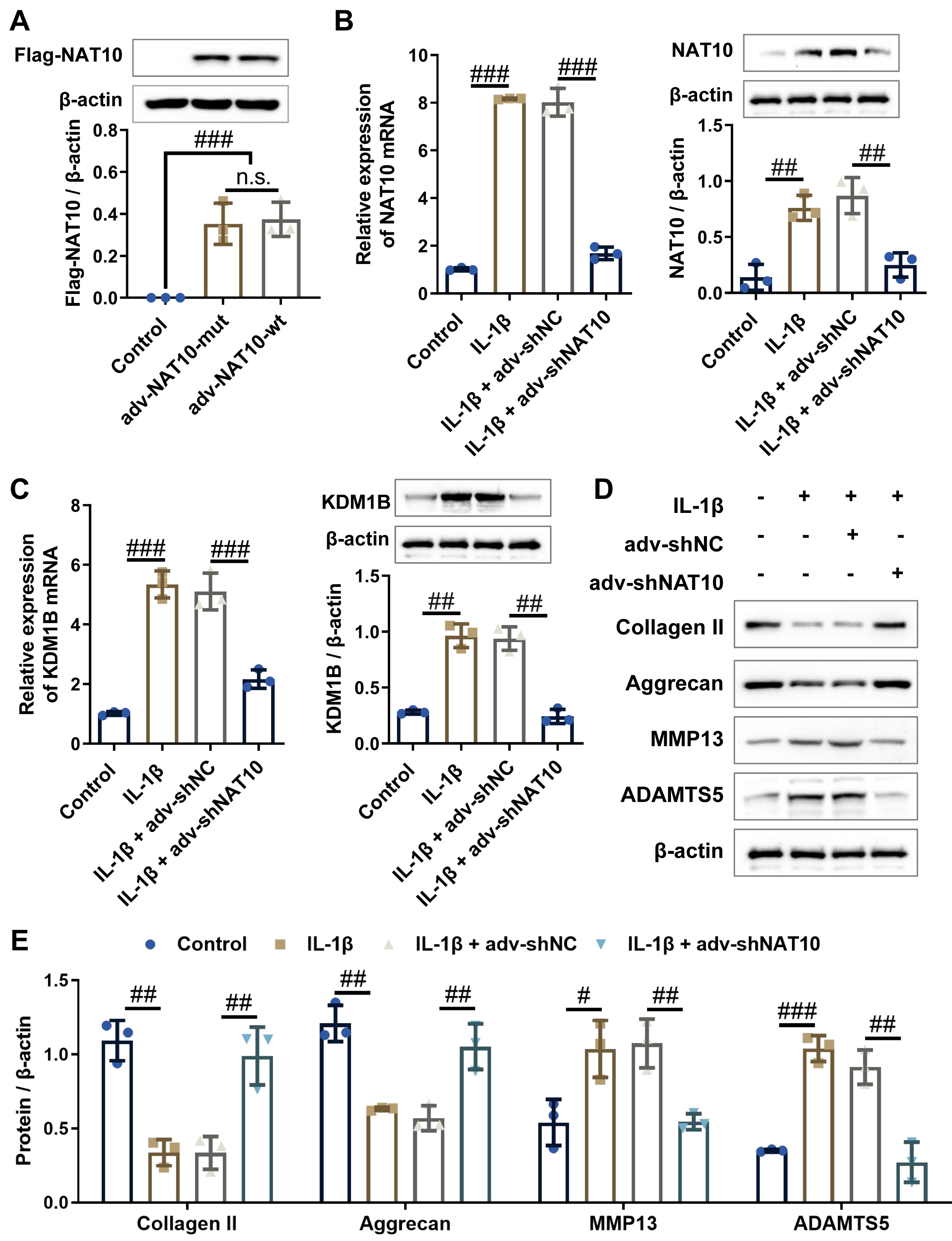

Supplement: Supplementary file 12 — Supplementary file6 (TIF 18.5 MB) [file 18_2025_5918_MOESM6_ESM.tif]

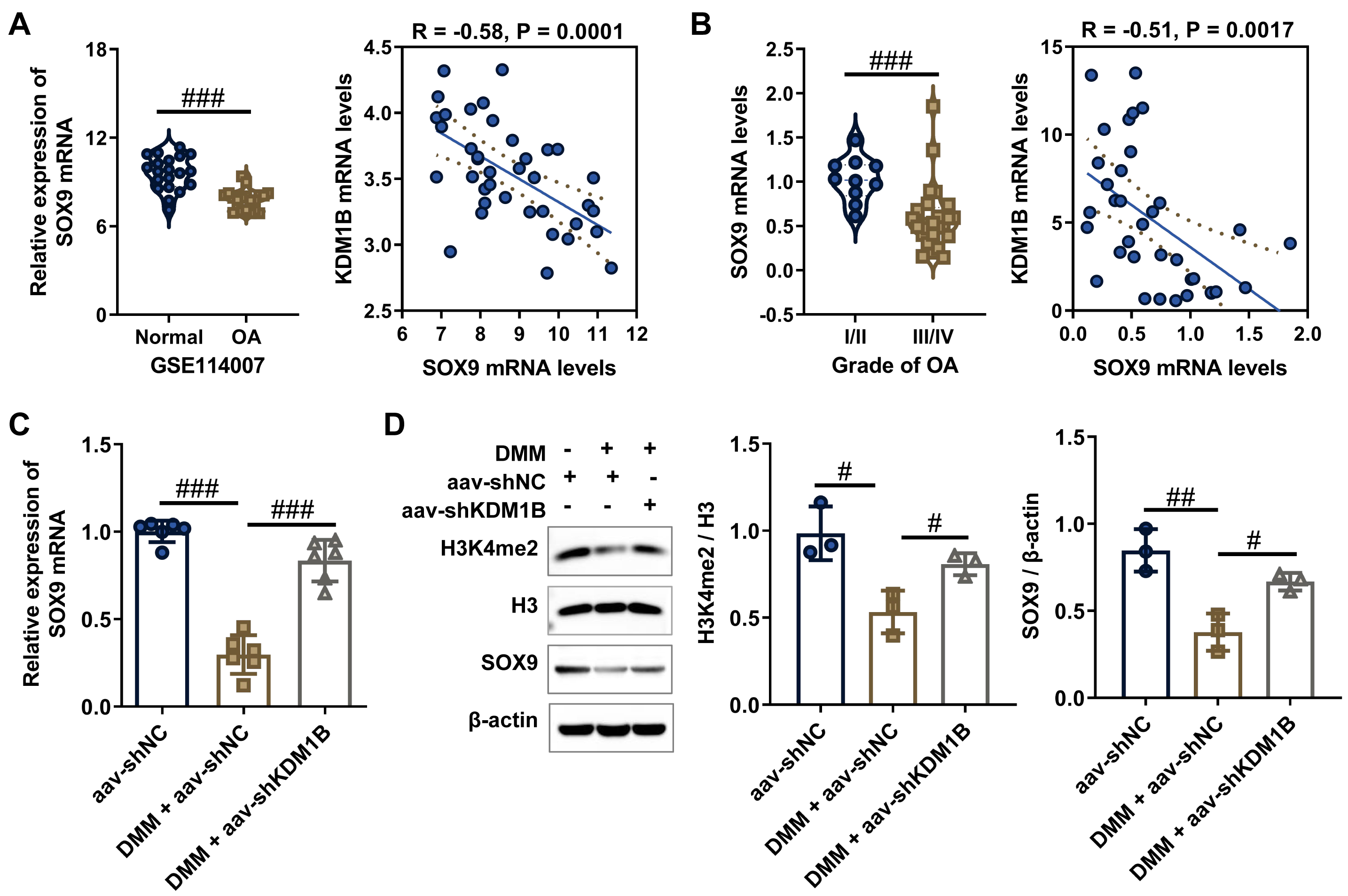

Supplement: Supplementary file 13 — The relationship between KDM1B and SOX9. A Analysis of the GSE114007 dataset revealed significant downregulation of SOX9 expression in human OA cartilage tissues (n = 20) compared to normal controls (n = 18). And Spearman correlation analysis further demonstrated a significant inverse correlation between SOX9 and KDM1B expression levels. B RT-qPCR analysis of human OA cartilage specimens revealed significantly downregulated SOX9 mRNA levels in grade III/IV tissues (n = 25) compared to grade I/II tissues (n = 10). And Spearman correlation analysis further demonstrated a significant inverse correlation between SOX9 and KDM1B expression levels. C The mRNA expression levels of SOX9 in cartilage tissue of OA mice treated with aav-shKDM1B were evaluated by RT-qPCR. D The protein expression levels of SOX9 and H3K4me2 in the indicated cartilage tissue were evaluated by western blot. Normal, non-OA cartilage; OA, osteoarthritis cartilage; DMM, destabilizing the medial meniscus-induced OA mice; aav-shNC, negative control short hairpin RNA adeno-associated virus; aav-shKDM1B, KDM1B short hairpin RNA adeno-associated virus. N = 3 ~ 6, #p < 0.05, ##p < 0.01, and ###p < 0.001 (PNG 682 KB) [file 18_2025_5918_Fig15_ESM.png]

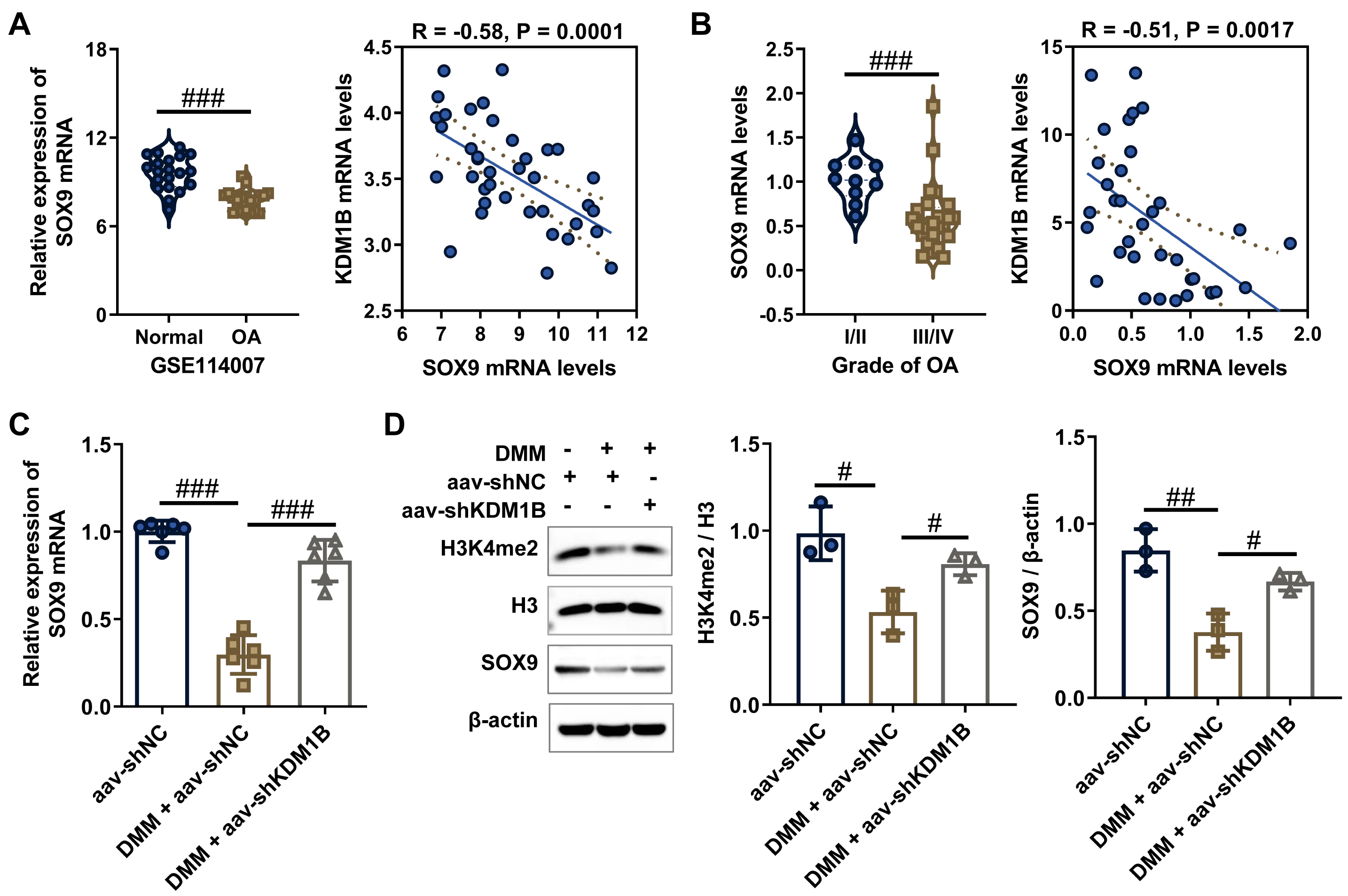

Supplement: Supplementary file 14 — Supplementary file7 (TIF 15.6 MB) [file 18_2025_5918_MOESM7_ESM.tif]

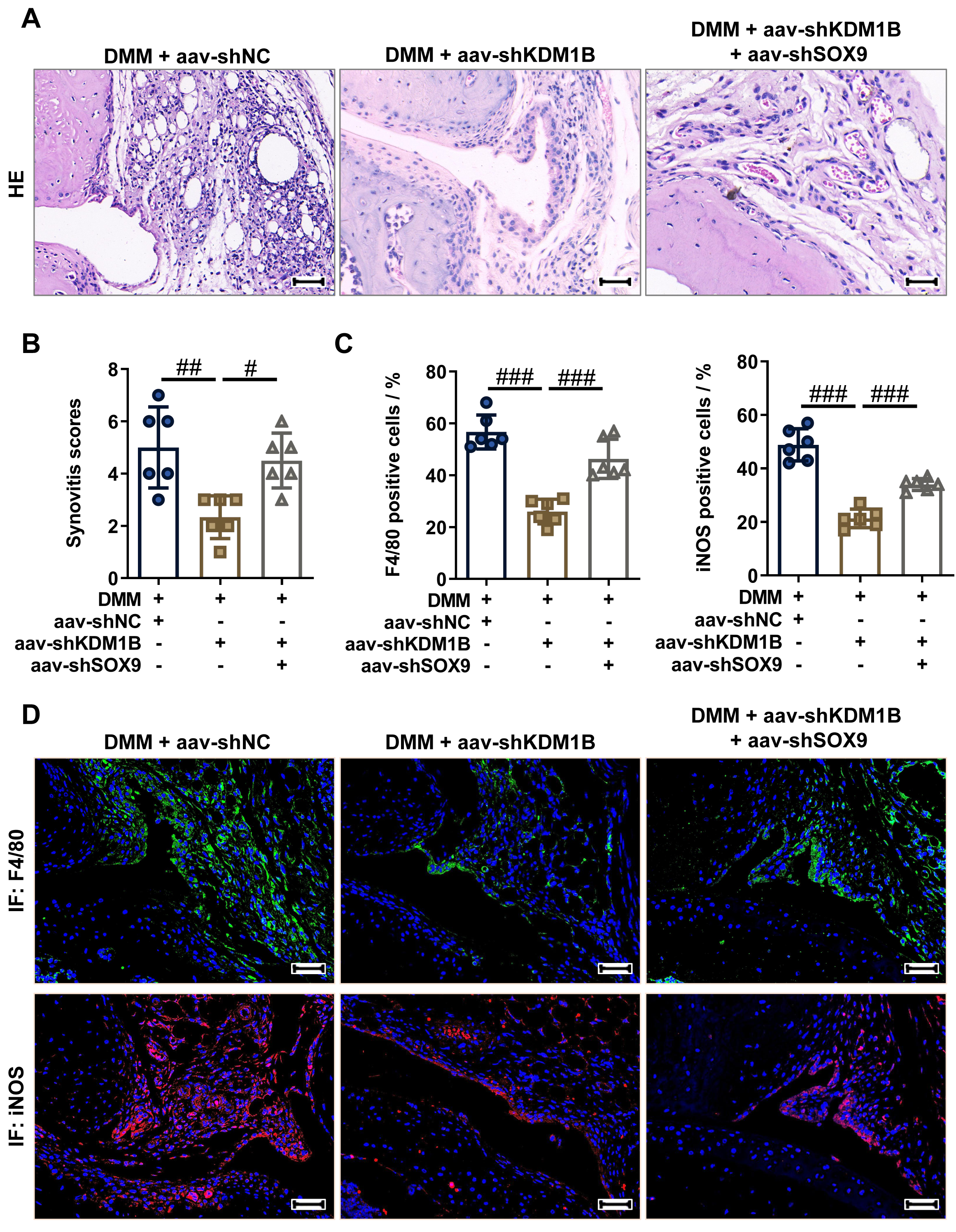

Supplement: Supplementary file 15 — KDM1B knockdown rescues experimental synovitis in DMM-induced OA mice through SOX9 upregulation. A Representative images of H&E staining in the indicated synovial tissues. Scale bars = 50μm. B Synovium scores was measured in synovial tissue from the indicated mice. C Quantitative immunofluorescence analysis of synovial F4/80+ macrophage infiltration and iNOS+ M1 polarization across experimental mouse cohorts. D Representative immunofluorescence micrographs of synovial membrane sections stained for macrophage marker F4/80 (green) and M1 polarization marker iNOS (pink). Nuclei counterstained with DAPI (blue), Scale bar = 50μm. DMM, destabilizing the medial meniscus-induced OA mice; aav-shNC, negative control short hairpin RNA adeno-associated virus; aav-shKDM1B, KDM1B short hairpin RNA adeno-associated virus; aav-shSOX9, SOX9 short hairpin RNA adeno-associated virus. N = 6, #p < 0.05, ##p < 0.01, and ###p < 0.001 (PNG 5.00 MB) [file 18_2025_5918_Fig16_ESM.png]

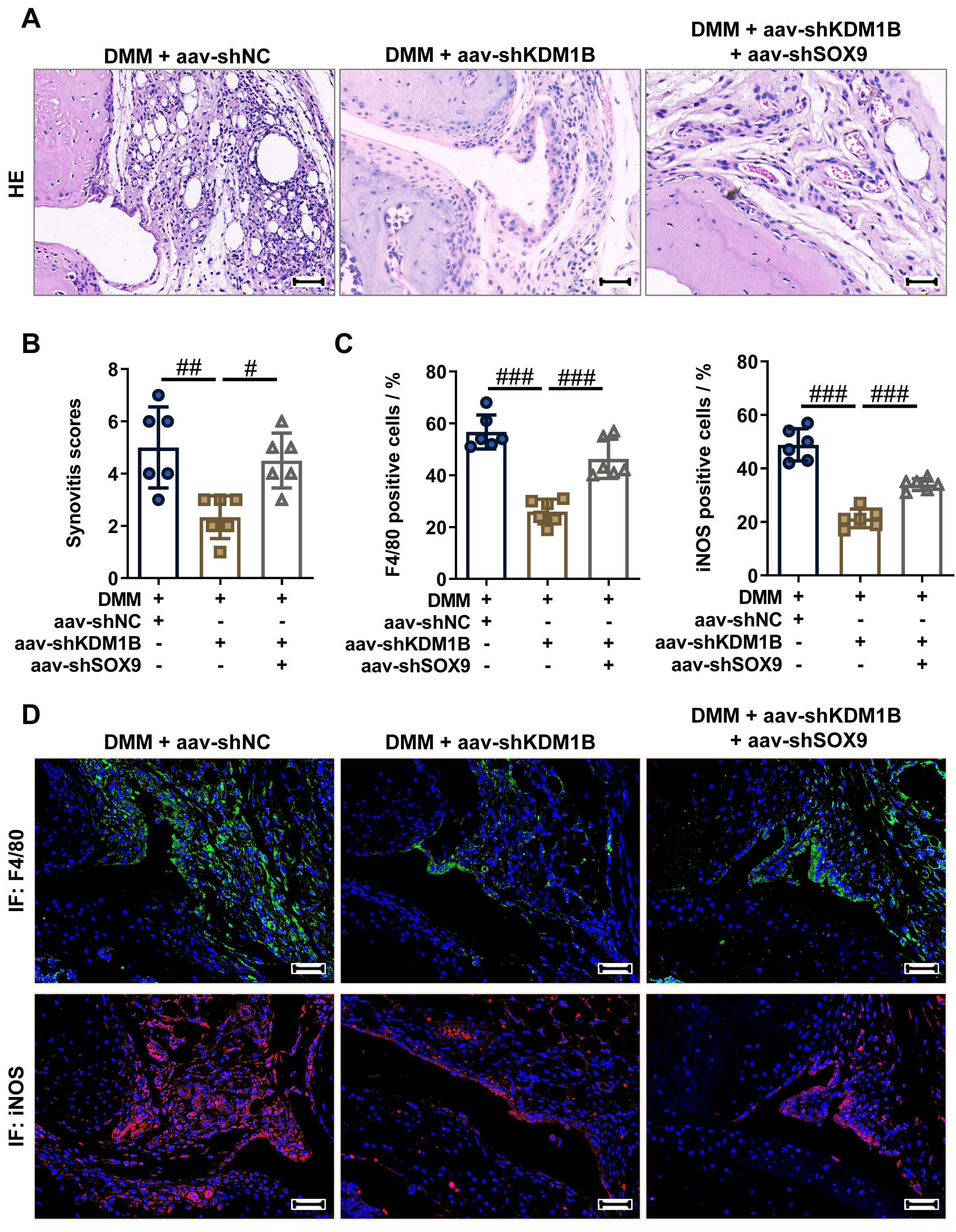

Supplement: Supplementary file 16 — Supplementary file8 (TIF 19.8 MB) [file 18_2025_5918_MOESM8_ESM.tif]
