# Supplementary material for: NAT10-mediated ac4C modification of KDM1B drives osteoarthritis progression through epigenetic suppression of SOX9
Source: Cell Mol Life Sci. 2025 Nov 26;82(1):422. doi: 10.1007/s00018-025-05918-z (PMC12647510; doi:10.1007/s00018-025-05918-z)
Supplement: Supplementary file 17 — Supplementary file4 (DOCX 30.4 KB) [file 18_2025_5918_MOESM9_ESM.docx]

| \| **Supplementary Table 1** All sequences of shRNAs and their negative control used in this study. \| \| \| --- \| --- \| \| Names \| Sequences (5’-3’) \| \| shKDM1B(human) \| CCGGTTAACAACCCAGTAGCATTAACTCGAGTTAATGCTACTGGGTTGTTAATTTTTGAATT \| \| shKDM1B(mouse) \| CCGGCCACTACTACAGAAGCCACAACTCGAGTTGTGGCTTCTGTAGTAGTGGTTTTTGAATT \| \| shNAT10(human) \| CCGGGCAATTGTACACAGTGACTATCTCGAGATAGTCACTGTGTACAATTGCTTTTTGAATT \| \| shSOX9(human) \| CCGGACCTTCGATGTCAACGAGTTTCTCGAGAAACTCGTTGACATCGAAGGTTTTTTGAATT \| \| shNC \| CCGGTCCTAAGGTTAAGTCGCCCTCGCTCGAGCGAGGGCGACTTAACCTTAGGTTTTTGAATT \|   **Supplementary Table 2** All primer sequences used in the present study. | | |
| --- | --- | --- | --- | --- | --- | --- | --- | --- | --- | --- | --- | --- | --- | --- | --- | --- |
| Symbol | Sequencing （5’-3’） | Product length |
| KDM1B (human) | Forward primer, CACGGGGGAGGACAAAGAAA | 235 |
|  | Reverse primer, CATCGGGAGGTGTAGCCATT |  |
| KDM1B (mouse) | Forward primer, CAAAGCAATGGCCGCATCT | 178 |
|  | Reverse primer, GCCTGCCTTTTCACATTTCCG |  |
| SOX9 (human) | Forward primer, GGAAGTCGGTGAAGAACGGG | 230 |
|  | Reverse primer, CTCTCGCTTCAGGTCAGCC |  |
| SOX9 (mouse) | Forward primer, CTACTCCACCTTCACTTACAT | 325 |
|  | Reverse primer, CAGCAATCGTTACCTTCCT |  |
| NAT10 (human) | Forward primer, CATACGCTAGGGGCAGTCAG  Reverse primer, TCTTTGCCGCTCAGCTACTC | 189 |
|  |  |  |
| GAPDH (human) | Forward primer, AAGTATGACAACAGCCTCAAG | 101 |
|  | Reverse primer, TCCACGATACCAAAGTTGTC |  |
| GAPDH (mouse) | Forward primer, AAGAGGGATGCTGCCCTTAC | 119 |
|  | Reverse primer, AAGAGGGATGCTGCCCTTAC |  |
| HPRT1 (human) | Forward primer, GGCGTCGTGATTAGTGATGA | 173 |
|  | Reverse primer, GCTACAATGTGATGGCCTCC |  |
| HPRT1 (mouse) | Forward primer, ACCCTCTGGTAGATTGTCGC | 206 |
|  | Reverse primer, AATCGAGAGCTTCAGACTCGT |  |
| ChIP_SOX9 pro#1 | Forward primer, AAGCCAGAGCAGTTAGCA | 271 |
|  | Reverse primer, GGGAGTCAATGGAAAGCAA |  |
| ChIP_SOX9 pro#2 | Forward primer, TGGATTATTACGGAGGAACAG | 308 |
|  | Reverse primer, CTCTCGGAATGCCAGAATT |  |
| ChIP_SOX9 pro#3 | Forward primer, AGTGCCACAATCCTCCTC | 108 |
|  | Reverse primer, ATACTCCGCCTCACCTTAG |  |

**Supplementary Table 3** Information on antibodies used for western blot in this study.

| Name | Catalog Number | Source | Dilution factor |
| --- | --- | --- | --- |
| Anti-KDM1B antibody | ab198080 | Abcam | 1:1000 |
| Anti-SOX9 antibody | ab155279 | Abcam | 1:1000 |
| Anti-NAT10 antibody | ab194297 | Abcam | 1:2000 |
| Anti-ADAMTS5 antibody | ab41037 | Abcam | 1:250 |
| Anti-MMP13 antibody | ab39012 | Abcam | 1:2500 |
| Anti-Collagen II | ab188570 | Abcam | 1:2000 |
| Anti-Aggrecan antibody | ab31639 | Abcam | 1:1000 |
| Anti-Ki-67 antibody | ab1667 | Abcam | 1:1500 |
| Anti-p21 antibody | ab109199 | Abcam | 1:5000 |
| Anti-p27 antibody | ab32034 | Abcam | 1:1000 |
| [Anti-beta Actin antibody](https://www.abcam.cn/products/primary-antibodies/beta-actin-antibody-mabcam-8226-loading-control-ab8226.html) | ab8226 | Abcam | 1 µg/mL |
| [Rabbit Anti-Mouse IgG](https://www.abcam.cn/products/secondary-antibodies/rabbit-mouse-igg-hl-hrp-ab6728.html) | ab6728 | Abcam | 1:10000 |
| [Goat Anti-Rabbit IgG](https://www.abcam.cn/products/secondary-antibodies/goat-rabbit-igg-hl-hrp-ab6721.html) | ab6721 | Abcam | 1:2000 |
